# Evaluation of health care professionals’ knowledge, attitudes, practices and barriers to pharmacovigilance and adverse drug reaction reporting: A cross-sectional multicentral study

**DOI:** 10.1371/journal.pone.0285811

**Published:** 2023-05-24

**Authors:** Zakir Khan, Yusuf Karatas, Syed Muhammad Hamid

**Affiliations:** 1 Department of Medical Pharmacology, Faculty of Medicines, Çukurova University, Adana, Türkiye; 2 Faculty of Medicines, Balcali Hospital, Adana, Türkiye; 3 Department of Community Medicine, Khyber Medical College, Peshawar, Pakistan; University of Gondar, ETHIOPIA

## Abstract

**Background:**

Healthcare professionals’ involvement and reporting of adverse drug reactions are essential for the success of a pharmacovigilance program. The aim of this study was to assess healthcare professionals (medical doctors, pharmacists, nurses, dentists, midwives, and paramedics) current knowledge, attitude, practices, and barriers regarding pharmacovigilance and adverse drug reactions reporting in multicentral healthcare settings.

**Methods:**

A cross-sectional face-to-face survey was conducted among currently working healthcare professionals in various hospitals in ten districts of Adana province, Türkiye from March to October 2022. A self-administered, pretested questionnaire (Cronbach’s alpha = 0.894 for knowledge, attitudes and practices variables) was used for data collection. The questionnaire’s final draft included five sections (sociodemographic/general information, knowledge, attitude, practices, and barriers) with 58 questions. The collected data was analyzed in SPSS (version 25) using descriptive statistics, the chi-square test, and logistic regression.

**Results:**

Of the total 435 distributed questionnaires, 412 completed the entire questionnaire, yielding a 94% response rate. The majority of healthcare professionals (60.4%; n = 249) had never received pharmacovigilance training. Among healthcare professionals 51.9% (n = 214), 71.1% (n = 293) and 92.5% (n = 381) had poor knowledge, positive attitudes and poor practices, respectively. Only 32.5% of healthcare professionals kept the record of an adverse drug reaction and only 13.1% reported adverse drug reactions. The profession (medical doctors, pharmacists, nurses, dentists, midwives, and paramedics) of healthcare professionals and a lack of training were predictors of poor adverse drug reaction reporting (p < 0.05). A statistically significant difference in healthcare professionals and knowledge, attitude and practices scores was also observed (p < 0.05). The main barriers which were supposed to discourage adverse drug reactions reporting by the healthcare professionals were higher workload (63.8%) followed by thinking that a single adverse drug reaction report makes no impact (63.6%) and lack of a professional atmosphere (51.9%).

**Conclusion:**

In the current study, most healthcare professionals had poor knowledge and practice, but they had a positive attitude toward pharmacovigilance and adverse drug reactions reporting. Barriers to under-reporting of adverse drug reactions were also highlighted. Periodic training programs, educational interventions, systematic follow-up of healthcare professionals by local healthcare authorities, interprofessional links between all healthcare professionals, and the implementation of mandatory reporting policies are critical for improving healthcare professionals knowledge, practices, patient safety and pharmacovigilance activities.

## Introduction

The World Health Organization (WHO) defines pharmacovigilance (PV) as "the science and activities related to the detection, understanding, and prevention of side effects or other drug-related problems" [1]. Globally, PV is an important clinical discipline for ensuring appropriate medication use and patient safety [2, 3]. PV is primarily focused on adverse drug reactions (ADRs), which can occur when a drug is administered incorrectly to a patient [4]. ADR is defined as any adverse reaction to an unwanted and harmful drug at normal dosages used for disease prevention, diagnosis, therapy, or modifying physiological disorder function [2]. ADR is a serious issue that contributes to increased morbidity and mortality worldwide [3]. It is documented that 5% of patients were hospitalized as a result of ADR, with another 5% experienced ADR during hospitalization [5]. ADR accounts for 197,000 fatalities annually in the European Union [6]. ADRs also increased the financial burden on patients. The total cost of hospitalization in the United States (US) following ADRs in the intensive care unit (ICU) and the non-ICU ward is estimated to be 19,685 US dollars and 13,994 US dollars, respectively [7]. According to reports, each patient’s treatment for an ADR is expected to cost on average 9,491 US dollars [7, 8]. Therefore, timely ADR monitoring and spontaneous reporting is essential for patient safety and reducing the financial burden on the healthcare system.

In PV, spontaneous ADR reporting schemes have been a significant source of medical data [9]. The spontaneous reporting of ADRs is the most crucial technique for enhancing PV data on drugs that were released into the market with limited premarketing safety information [10]. This method can help to prevent new medicine tragedies and improve pharmaceutical product safety labeling [11]. The success of a PV program is dependent on healthcare professionals (HCP) participation and reporting of ADRs [9, 10]. Doctors, pharmacists, nurses and other paramedics bear an enormous obligation to report ADRs and strengthen the PV systems [9, 10, 12, 13]. However, spontaneous ADR schemes are linked to low levels of reporting. A systematic review of studies conducted in the European Union found a widespread under-reporting of ADR (median rate of 94% under-reporting) among HCPs [14]. It is the responsibility of all HCPs to recognize, record and report ADRs, and their assistance is also necessary for the early recognition of ADR [15]. However, numerous reasons affect reporting of ADRs by HCP, such as ignorance, ambiguity regarding the ADR, and difficulty in comprehending the reporting method [9, 16]. Moreover, strong evidence also exists that many ADRs including, serious or severe ADRs are underreported to spontaneous reporting systems [14]. Therefore, it may take a longer time for regulatory actions to remove drugs from the market with an unacceptable safety profile due to the low rate of ADR reporting [15].

Findings from international studies revealed that ADR reporting is related to HCPs’ knowledge, attitudes, and practices (KAPs) [4, 12, 15, 16]. It is important to understand HCPs’ KAP and perceived barriers to PV and ADR [9, 13, 15]. Periodic evaluation of the HCPs’ KAP toward PV and ADR reporting can aid in the development of strategies to improve reporting schemes and ensure patient safety [4, 10, 17]. Most countries have formal policies in place to provide training on PV and assist HCPs to notify their national drug regulatory agency or the pharmaceutical manufacturer about suspected ADRs encountered in clinical practice [4, 5, 11, 12, 17, 18]. In Türkiye, the first PV regulation was published in 2005, and the most recent one went into effect in 2014 [19]. The Turkish pharmacovigilance center (TÜFAM) is responsible for implementing PV in Türkiye [20]. The PV program in Türkiye is still in its infancy, only a few ADRs have been reported to the centre since the implementation of the PV system. According to recent data conveyed by Türkiye to the WHO-Upsala Monitoring center (WHO-UMC), the reported ADRs per million population/year for the years 2017, 2018, 2019, 2020, 2021 and 2022 were 84, 94, 99, 89, 91 and 105 respectively [21, 22]. However, still, Türkiye ’s reporting rate of ADR is significantly lower than the WHO-UMC’s suggested ideal value (200 ADR reports per million/year) [21, 23–25]. More proactive initiatives and policy implementations are needed to encourage PV knowledge and ADR reporting behaviors among HCPs to increase the reporting rate of ADRs in Türkiye [9, 10, 20].

A recent systematic review found a significant KAP gap between PV activities and HCPs in Türkiye [9]. Few studies conducted on a national scale revealed a lack of ADR awareness and reporting among Turkish HCPs [9, 10]. However, these studies are insufficient to fully comprehend the concept of PV among various Turkish HCPs, as well as the reasons for under-reporting in Türkiye [9]. Periodic understanding of the KAP of HCPs regarding PV and ADR reporting is crucial for the improved ADR reporting rate because numerous studies have shown that optimizing PV KAP is essential for creating improvement strategies for better ADR reporting systems [4, 12, 17, 26, 27]. Therefore, the aim of this study was to assess the current knowledge, attitudes, and practices (KAP) and barriers of HCPs (medical doctors, pharmacists, nurses, dentists, midwives, and paramedics) regarding PV and ADR reporting in multicentral healthcare settings in the Adana province, Türkiye.

## Materials and methods

### Study design, population, and study setting

A cross-sectional face-to-face questionnaire-based survey was conducted in Adana, Türkiye, targeting currently working HCPs in various healthcare settings. This study was carried out among HCPs working in different hospitals in ten districts of Adana (Cukurova, Seyhan, Yuregir, Saricam, Ceyhan, Kozan, Imamoglu, Karaisali, Karatas, and Yumurtalik) during March to October 2022. Participants in the study were full-time registered HCPs with at least one year of work experience. Healthcare students on a traineeship, HCPs with less than one year of professional experience, and unwilling to participate were excluded.

### Sampling technique and sample size

HCPs who were available on the day of data collection were selected by using a convenience sampling strategy. Participants for convenience sampling are chosen based on their ease of access and proximity to the study site [28]. The ministry of health, provincial health directorate (Sağlik Bakanliği, Adana il sağlik müdürlüğü) office in Adana was contacted, and a list of the registered working HCPs was obtained [29]. As per the ministry of health, Adana, the number of registered medical doctors, dentists, pharmacists, nurses, midwives, and paramedics were 2513, 267, 109, 4980, 700, and 1200, respectively. Using a single population proportion formula, the minimal sample size needed was calculated. The total number of registered HCPs was 9769. The minimum required sample size was 370, as per the Raosoft sample size calculator [30]. The response distribution was assumed to be 50%, the error margin was 5% and the confidence level was set at 95% for the estimation of a sample. All HCPs were first divided into groups based on the type of profession, and this list served as the sampling frame. The questionnaire was distributed to 435 HCPs to ensure reliability and account for any missing data or response rate.

### Questionnaire instrument

A comprehensive literature search was conducted to design a questionnaire using keywords and medical subject headings (MeSH) terms such as "knowledge, attitude, and practice; pharmacovigilance; adverse drug reaction reporting by healthcare professionals; barriers to pharmacovigilance and adverse drug reaction reporting." A self-reported questionnaire was developed after a literature review [9, 12, 15, 17, 31–33]. The questionnaire was translated using the direct translation method [34, 35] from the source language (English) to the target language (Turkish, which is the official language of Türkiye) by two qualified and experienced researchers who spoke Turkish as well as English and whose native or inborn language was Turkish. Both researchers were aware of the aims and objectives of the questionnaire. Furthermore, the questionnaire was adopted and modified as per WHO PV readings guidelines [36].

After that, the questionnaire was reviewed by three academic expert researchers to determine its suitability, consistency, adequacy, and validity. The questionnaire was tested in Cukurova University’s faculty of medicine, department of medical pharmacology and affiliated hospital (Balcali Hastanesi: Balcali hospital) with 30 HCPs (5 doctors, 5 dentists, 5 pharmacists, 5 nurses, 5 midwives, and 5 paramedics) to determine comprehension of the language used in the questionnaire and its suitability for accurately measuring the variables under observation. HCPs selected for the pilot study were excluded from the study’s final sample. Several changes were made based on feedback from the pre-tested questionnaire, including changes to the KAP variables, the removal of repetitive questions, and the inclusion of question related to PV training. Furthermore, the internal consistency "reliability" of the items was calculated using Cronbach’s alpha (a value of more than 7 is regarded as acceptable to indicate a good fit for internal consistency). Using the Statistical Package for the Social Sciences (SPSS: Chicago, IL, U.S.A, Version 25.0), the questionnaire items’ internal consistency for knowledge, attitude, and practices was determined as Cronbach’s alpha value 0.894 (Cronbach’s Alpha based on standardized items = 0.883). The questionnaire’s reliability of each section followed: knowledge (0.797), attitude (0.908) and practice (0.741). Following these changes, principal investigators distributed the final questionnaire to eligible HCPs in various healthcare settings.

### Data collection tool (questionnaire)

The data was gathered using a structured questionnaire. The questionnaire’s final draft included five sections and 58 questions (S1 File).

1. The first section included sociodemographic and general information (Gender, age, profession, workplace, name of working district, experience, and prior training on PV)
2. The second part contained two sections. The first consisted of 1 to 8 closed-ended questions (Yes/No), and the second consisted of 1 to 8 multiple-choice items about PV and ADR reporting knowledge.
3. The third section included 9 Likert-scale questions (5 points: strongly disagree, disagree, uncertain, agree, strongly agree) about HCPs’ attitudes toward PV and ADR reporting.
4. The fourth section included eight closed-ended questions (Yes/No) about HCPs’ PV and ADR reporting practices.
5. The final section included 18 closed-ended questions (Yes/No/I don’t know) to understand the barriers and reasons regarding the PV and ADRs reporting system.

### Statistical analysis

First, the final collected information was reviewed for discrepancies and missing data. The data from each questionnaire was then entered into SPSS for statistical analysis. Various statistical measuring techniques used in this study are listed below;

### Measuring techniques

**Descriptive statistics:** Frequency, percentage, mean and standard deviation were used to analyze the data.

**Modified Bloom’s cut-off point criteria for KAP score:** The overall percent score cut-off was determined using modified Bloom’s cut-off point criteria, which had previously been used in related studies [26, 27]. For the knowledge section, we chose 8 questions (1–8 from section 2a), with each correct response (yes:1) receiving a score of one and incorrect response (no:0) receiving a zero score. The total score for all items was 8. HCPs were classified as "good knowledge" if the score ranged from 75%–100% (6–8 points), "moderate knowledge" if the score ranged from 50–74% (4–5 points), and "poor knowledge" if the score ranged less than 50% (less than 4 points) [26, 27]. Respondents were asked to rate how much they agreed or disagreed with various statements including, "strongly disagree," "disagree," "uncertain," "agree," and "strongly agree" on a 5-point Likert scale attitude section (9 questions), with values ranging from 1 to 5. When all of the items are added together, the total score is 45. The original Bloom’s cut-off point was used to categorize overall attitude levels [26, 27]. A "positive attitude" was defined as a score of 80–100% (36–45 points), a "moderate attitude" as a score of 60–79% (27–35 points), and a "negative attitude" as a score of less than 60% (26 points). There were also eight yes/no questions about practice-related behavior. The "yes" response received a 1, while the "no" response received a 0. Using Bloom’s original cutoff point, the total practice score was classified as "good practice" if it was 80–100 percent (7–8 points) and "poor practice" if it was less than 80 percent (6 points) [26, 27].

**Chi-square test analysis:** Chi-square test analysis was used to evaluate the results of HCPs responses for comparison purposes.

**Logistic regression:** Univariate and multivariate logistic regression tests were used to identify the factors associated with ADR reporting. The ADR reporting (ever sent a suspected ADR report to a hospital/national PV center) was the dependent variable, while gender, profession, workplace, district, experience, and PV training were included as independent variables. A p-value of less than 0.05 is considered statistically significant.

### Ethics approval

This study was approved by the Cukurova University Bioethics committee (Meeting number: 11; reference number: 50243401/2021-6; Dated: 21/05/2021). In addition, ethical approvals were also taken from the ministry of health, provincial health directorate (Sağlik Bakanliği, Adana il sağlik müdürlüğü) office in Adana (Reference number: E-96172664-604.01.02 Dated: 26/11/2021). Both informed and written consent was sought from each participant by using a consent form before they were enrolled in the study. Survey confidentiality and anonymity were assured to all enrolled participants.

## Results

Of the total 435 distributed questionnaires, 412 (150 nurses, 105 doctors, 70 paramedics, 38 dentists, 29 midwives, and 20 pharmacists) completed the entire questionnaire, yielding a 94% response rate. Twenty-three were excluded (missing information = 8, lack of time = 11, and having less than 1 year of experience = 4) from the final analysis. Females (63.8%) were more than males (36.2%). The mean (± SD) age of the HCPs was 36.7 (± 8.97) years, ranging from 22 to 61 years. Among the respondents’ institutions, 61.4% (n = 253) were government hospitals, followed by 112 control command centers (n = 77; 18.7%), university hospitals (n = 32; 7.8%), and private hospitals (n = 27; 6.6%). The majority of the HCPs were from Yuregir (28.4%), Kozan (22.3%), and Cukurova (15.8%) districts. A higher proportion of the HCPs (34.2%; n = 141) had been working for more than 15 years. Most of the HCPs (60.4%; n = 249) did not attend PV training previously. A statistically significant difference was also observed between profession for gender (p-value = 0.001), hospital type (p-value = 0.001), district of working (p-value = 0.001), experience (p-value = 0.004), and PV training (p-value = 0.001) **(Table 1)**.

**Table 1:** Demographic variables and general information of HCPs (n = 412).

Variables	Doctor (n = 105)	Dentist (n = 38)	Pharmacist (n = 20)	Nurse (n = 150)	Midwife (n = 29)	Paramedic (n = 70)	Total (n = 412)	*P-value
Gender								0.001
Female	40 (38.1)	18 (47.4)	12 (60)	121 (80.7)	27 (93.1)	45 (64.3)	263 (63.8)	
Male	65 (61.9)	20 (52.6)	8 (40)	29 (19.3)	2 (6.9)	25 (35.7)	149 (36.2)	
Workplace								0.001
Government/Public hospital	61 (58.1)	35 (92.1)	16 (80)	114 (76)	26 (89.7)	1 (1.4)	253 (61.4)	
112 Control command center	9 (8.6)	0 (0)	0 (0)	2 (1.9)	0 (0)	66 (94.3)	77 (18.7)	
University hospital	16 (15.2)	1 (2.6)	1 (5)	13 (8.7)	1 (3.4)	0 (0)	32 (7.8)	
Private hospital	10 (9.5)	2 (5.2)	3 (15)	10 (6.6)	0 (0)	2 (2.8	27 (6.6)	
Integrated district hospital	5 (4.8)	0 (0)	0 (0)	5 (3.3)	2 (6.9)	1 (1.4)	13 (3.2)	
Family healthcare center	4 (3.8)	0 (0)	0 (0)	6 (4)	0 (0)	0 (0)	10 (2.4)	
District								0.001
Yuregir	35 (33.3)	17 (44.7)	2 (10)	30 (20)	1 (3.4)	32 (45.7)	117 (28.4)	
Kozan	26 (24.8)	8 (21)	4 (20)	41 (27.3)	13 (44.8)	0 (0)	92 (22.3)	
Cukurova	14 (13.3)	9 (23.7)	10 (50)	18 (12)	2 (6.9)	12 (17.1)	65 (15.8)	
Karaisali	6 (5.7)	2 (5.2)	2 (10)	26 (17.3)	6 (20.7)	1 (1.4)	43 (10.4)	
Seyhan	6 (5.7)	1 (2.6)	0 (0)	7 (4.7)	0 (0)	22 (31.4)	36 (8.7)	
Ceyhan	7 (6.7)	1 (2.6)	0 (0)	10 (6.7)	0 (0)	1 (1.4)	19 (4.6)	
Saricam	5 (4.7)	0 (0)	2 (10)	8 (5.3)	1 (3.4)	2 (2.8)	18 (4.4)	
Imamoglu	1 (0.9)	0 (0)	0 (0)	5 (3.3)	5 (17.2)	0 (0)	11 (2.7)	
Yumurtalik	5 (4.7)	0 (0)	0 (0)	1 (0.7)	0 (0)	0 (0)	6 (1.5)	
Kararatas	0 (0)	0 (0)	0 (0)	4 (2.7)	1 (3.4)	0 (0)	5 (1.2)	
Working experience								0.04
1–5 years	29 (27.6)	9 (23.7)	8 (40)	26 (17.3)	7 (24.1)	7 (10)	86 (20.9)	
6–10 years	17 (16.2)	12 (31.6)	2 (10)	28 (18.7)	3 (10.3)	25 (35.7)	87 (21.1)	
11–15 years	24 (22.8)	7 (18.4)	4 (20)	36 (24)	5 (17.2)	22 (31.4)	98 (23.8)	
More than 15	35 (33.3)	10 (26.3)	6 (30)	60 (40)	14 (48.3)	16 (22.8)	141 (34.2)	
PV training								0.001
Yes	30 (28.6)	15 (39.5)	11 (55)	82 (54.7)	19 (65.5)	6 (8.6)	163 (39.6)	
No	75 (71.4)	23 (60.5)	9 (45)	68 (45.3)	10 (34.5)	64 (91.4)	249 (60.4)	

*P-value: Chi square test, PV: Pharmacovigilance

### Knowledge about PV and ADR (close-ended questions)

About, 68.2% (n = 281) of the respondents indicated that they were aware of the terms “PV” and “ADR”. Among all HCPs, the majority of the pharmacists (85%), midwives (82.8%), and medical doctors (73.3%) were aware of the PV term. The majority of pharmacists (85%), midwives (82.2%), nurses (74%), and medical doctors (68.6%) indicated knowing about the ADR term. Most of the participants (n = 268; 65%) did not know about the filling of the ADR reporting form. The higher proportion of dentists (84.2%), medical doctors (n = 87; 82.9%) and paramedics (82.9%) did not know about the filling of the ADR reporting form. One-fourth proportion (25%: n = 103) of the HCPs were aware regarding the International PV and ADR monitoring center. Of these, dentists (94.7%), midwives (82.8%), medical doctors (81%), and paramedics (74.3%) did not know about the International PV and ADR monitoring center however, pharmacists (70%) and nurses (29.3%) indicated their awareness about the center. More than half of the HCPs (57.8%; n = 238) were unable to explain TÜFAM expansion. Pharmacists (75%), midwives (65.5%), and paramedics (45.7%) were more able to define TÜFAM expansion as compared to nurses (44.7%), dentists (39.5%), and medical doctors (24.8%). Additionally, 51.9% (n = 214) of HCPs were aware of hospital PV contact points. The majority of the pharmacists (85%), midwives (69%), and nurses (68.7%) were aware of hospital PV. A statistically significant difference (p-value < 0.05) was also observed among most of the knowledge-related variables (close-ended questions) and HCPs professions (Table 2). The overall mean knowledge score was 3.47 ±2.37 (min-max = 0–8). More than half of the HCPs (n = 214; 51.9%) had poor knowledge. Among these, paramedics (71.4%), medical doctors (64.7%), and dentists (50%) had poor knowledge as compared to nurses (41.3%), midwives (37.9%), and pharmacists (20%). About, 24.5% (n = 101) and 23.5% (n = 97) of the HCPs had moderate and good knowledge, respectively. Pharmacists (65%) and nurses (31.3%) had more good knowledge of PV and ADRs than other HCPs. A statistically significant difference was also observed for HCPs’ professions and knowledge score (p-value = 0.00) (S1 Table).

**Table 2:** Knowledge (close-ended question) of HCPs about PV and ADRs.

Close-ended questions	Professions	Yes	No	*P-value
Awareness about PV	Medical doctor (n = 105)	77 (73.3)	28 (26.7)	0.001
	Dentist (n = 38)	24 (63.1)	14 (18.9)	
	Pharmacist (n = 20)	17 (85)	3 (15)	
	Nurse (n = 150)	109 (72.7)	41 (27.3)	
	Midwife (n = 29)	24 (82.8)	5 (17.2)	
	Paramedic (n = 70)	30 (42.9)	40 (57.1)	
	Total (412)	281 (68.2)	131 (31.8)	
Awareness about ADR	Medical doctor (n = 105)	72 (68.6)	33 (31.4)	0.001
	Dentist (n = 38)	20 (52.6)	18 (47.4)	
	Pharmacist (n = 20)	17 (85)	3 (15)	
	Nurse (n = 150)	111 (74)	39 (26)	
	Midwife (n = 29)	24 (82.8)	5 (17.2)	
	Paramedic (n = 70)	37 (52.9)	33 (47.1)	
	Total (412)	281 (68.2)	131 (31.8)	
Aware about the difference between ADR and AEs	Medical doctor (n = 105)	57 (54.3)	48 (45.7)	0.001
	Dentist (n = 38)	19 (50)	19 (50)	
	Pharmacist (n = 20)	15 (75)	5 (25)	
	Nurse (n = 150)	65 (43.3)	85 (56.7)	
	Midwife (n = 29)	8 (27.6)	21 (72.4)	
	Paramedic (n = 70)	12 (17.1)	58 (82.9)	
	Total (412)	176 (42.7)	236 (57.3)	
Read research publications and books on PV and ADR	Medical doctor (n = 105)	12 (11.4)	93 (88.6)	0.93
	Dentist (n = 38)	4 (10.5)	34 (89.5)	
	Pharmacist (n = 20)	7 (35)	13 (65)	
	Nurse (n = 150)	24 (16)	126 (84)	
	Midwife (n = 29)	3 (10.3)	26 (89.7)	
	Paramedic (n = 70)	8 (11.4)	62 (88.6)	
	Total (412)	58 (14.1)	354 (85.9)	
Knowledge about filling out the ADR reporting form	Medical doctor (n = 105)	18 (17.1)	87 (82.9)	0.001
	Dentist (n = 38)	6 (15.8)	32 (84.2)	
	Pharmacist (n = 20)	12 (60)	8 (40)	
	Nurse (n = 150)	78 (52)	72 (48)	
	Midwife (n = 29)	18 (62.1)	11 (37.9)	
	Paramedic (n = 70)	12 (17.1)	58 (82.9)	
	Total (412)	144 (35)	268 (65)	
Awareness of International PV and ADR monitoring center	Medical doctor (n = 105)	20 (19)	85 (81)	0.001
	Dentist (n = 38)	2 (5.3)	36 (94.7)	
	Pharmacist (n = 20)	14 (70)	6 (30)	
	Nurse (n = 150)	44 (29.3)	106 (70.7)	
	Midwife (n = 29)	5 (17.2)	24 (82.8)	
	Paramedics (n = 70)	18 (25.7)	52 (74.3)	
	Total (412)	103 (25)	309 (75)	
Able to explain TÜFAM expansion	Medical doctor (n = 105)	26 (24.8)	79 (75.2)	0.001
	Dentist (n = 38)	15 (39.5)	23 (60.5)	
	Pharmacist (n = 20)	15 (75)	5 (25)	
	Nurse (n = 150)	67 (44.7)	83 (55.3)	
	Midwife (n = 29)	19 (65.5)	10 (34.5)	
	Paramedic (n = 70)	32 (45.7)	38 (54.3)	
	Total (412)	174 (42.2)	238 (57.8)	
Awareness about hospital PV contact point.	Medical doctor (n = 105)	40 (38.1)	65 (61.9)	0.001
	Dentist (n = 38)	17 (44.7)	21 (55.3)	
	Pharmacist (n = 20)	17 (85)	3 (15)	
	Nurse (n = 150)	103 (68.7)	47 (31.3)	
	Midwife (n = 29)	20 (69)	9 (31)	
	Paramedic (n = 70)	17 (24.3)	53 (75.7)	
	Total (412)	214 (51.9)	198 (48.1)	

*P-value: Chi square test, PV: Pharmacovigilance, ADR: Adverse drug reaction, AEs: Adverse events, TÜFAM [English: Turkish pharmacovigilance center, Turkish: Türkiye Farmakovijilans Merkezi].

### Knowledge about PV and ADR (multiple choice questions: Only one response was allowed)

Most of the HCPs indicated that they heard the PV term for the first time during the “student stage” (35.2%; n = 145) followed by “training/continuing education programs” (29.4%; n = 121) and “in this survey” (21.8%; n = 90). Paramedics (52.9%) followed by dentists (23.7%) and nurses (18%) answered that they heard the PV term in this survey. Pharmacists (55%) and medical doctors (52.4%) stated that they heard the term PV when they were students. The terms ‘PV’ and “ADR” were correctly indicated by 71.4% (n = 294) and 69.7% (n = 287) of the participants. Pharmacists (85%) followed by midwives (82.7%) and medical doctors (77.1%) indicated the correct definition of PV. Similarly, a correct answer regarding the ADR definition was given by pharmacists (90%), midwives (79.3%), and medical doctors (79%) as compared to other groups of HCPs. More than half of the HCPs (n = 210; 51%) were aware that all ADRs (all serious ADRs, related to drugs, herbs, new drugs, vaccines, cosmetics, and old drugs) must be reported. However, 18.2% (n = 75) did not know about which ADRs must be reported. Among them, the majority of the midwives (65.5%) and pharmacists (65%) indicated a correct response that all ADRs should be reported. Moreover, most of the respondents indicated that a serious ADR must be reported to the national PV center within 7 days (n = 102; 24.8%) followed by 15 days (n = 68; 16.5%). Pharmacists (25%), midwives (20.7%), and paramedics (20%) respond with the right option that a serious ADR must be reported to the national PV center within 15 days. Nearly half of the participants (49.3%; 203) did not encounter ADR during their working experience. Most of the dentists (63.1%), nurses (58.7%), and paramedics (52.8%) reported that they did not encounter any ADR during their work experience. A statistically significant difference was also observed between professions for “first hear the PV term (p = 0.001), “appropriate ADR definition (p = 0.001), “which ADRs must be reported (p = 0.002), and “ADR-related patients encountered during work experience (p = 0.001)” while no statistical difference between professions for “most accurate definition of PV (p = 0.89) and “a serious ADR must be reported to the national PV center (p = 0.384) (Table 3).

**Table 3:** Multiple choice knowledge questions about PV and ADR among HCPs (n = 412).

*Multiple-choice questions	Doctor (n = 105)	Dentist (n = 38)	Pharmacist (n = 20)	Nurse (n = 150)	Midwife (n = 29)	Paramedic (n = 70)	Total	**P-value
**When did you first hear the PV term in?**								0.001
This survey	11 (10.5)	9 (23.7)	1 (5)	27 (18)	5 (17.2)	37 (52.9)	90 (21.8)	
Training/continuing education programs	16 (15.2)	14 (36.8)	7 (35)	60 (40)	12 (41.4)	12 (17.1)	121 (29.4)	
When I was a student	55 (52.4)	12 (31.6)	11 (55)	44 (29.3)	9 (31)	14 (20)	145 (35.2)	
In congress/meetings	15 (14.3)	0 (0)	1 (5)	6 (4)	0 (0)	4 (5.7)	26 (6.3)	
PV contact point	6 (5.7)	1 (2.6)	0 (0)	12 (8)	3 (10.3)	1 (1.4)	23 (5.6)	
Pharmaceutical company representative	2 (1.9)	2 (5.3)	0 (0)	1 (0.7)	0 (0)	2 (2.8)	7 (1.7)	
**Most accurate definition of PV?**								0.89
Activities related to the detection, assessment, understanding, and prevention of adverse drug effects.	81 (77.1)	25 (65.8)	17 (85)	102 (68)	24 (82.7)	45 (64.3)	294 (71.4)	
Detection of the type and frequency of ADRs after a drug has been marketed.	10 (9.5)	2 (5.3)	0 (0)	5 (3.3)	0 (0)	5 (7.1)	22 (5.3)	
The process of enhancing drug safety.	2 (1.9)	1 (2.6)	0 (0)	8 (5.3)	1 (3.4)	0 (0)	12 (2.9)	
The science of ADR monitoring in a hospital.	0 (0)	1 (2.6)	0 (0)	1 (0.7)	0 (0)	0 (0)	2 (0.5)	
None of the above.	0 (0)	0 (0)	0 (0)	0 (0)	0 (0)	0 (0)	0 (0)	
Do not know.	12 (11.4)	9 (23.7)	3 (15)	34 (22.7)	4 (13.8)	20 (28.6)	82 (19.9)	
**Appropriate definition of an ADR?**								0.001
Any undesirable effect of a drug that occurs at normal doses and under normal conditions of use.	83 (79)	24 (63.1)	18 (90)	98 (65.3)	23 (79.3)	41 (58.6)	287 (69.7)	
Adverse health outcomes associated with irrational/ inappropriate use of the drug.	11 (10.5)	2 (5.3)	0 (0)	17 (11.3)	4 (13.8)	1 (1.4)	35 (8.5)	
Injury caused by the use of substandard/counterfeit medications.	0 (0)	1 (2.6)	0 (0)	2 (1.3)	0 (0)	0 (0)	3 (0.7)	
Damage caused by a drug overdose.	1 (0.9)	2 (5.3)	0 (0)	0 (0)	0 (0)	6 (8.6)	9 (2.2)	
None of the above.	0 (0)	0 (0)	0 (0)	3 (2)	1 (3.4)	1 (1.4)	5 (1.2)	
Do not know.	10 (9.5)	9 (23.7)	2 (20)	30 (20)	1 (3.4)	21 (30)	73 (17.7)	
**Which ADRs must be reported?**								0.002
All serious ADRs.	30 (28.6)	9 (23.7)	5 (25)	57 (38)	4 (13.8)	15 (21.4)	120 (29.1)	
ADRs to herbal drugs.	0 (0)	1 (2.6)	0 (0)	0 (0)	0 (0)	0 (0)	1 (0.2)	
ADRs to new drugs.	1 (0.9)	0 (0)	0 (0)	2 (1.3)	0 (0)	0 (0)	3 (0.7)	
ADRs to vaccines.	0 (0)	0 (0)	0 (0)	1 (0.7)	0 (0)	1 (1.4)	2 (0.5)	
ADRs due to cosmetic use.	0 (0)	0 (0)	0 (0)	0 (0)	0 (0)	0 (0)	0 (0)	
Unknown ADRs to old drugs.	0 (0)	0 (0)	0 (0)	0 (0)	1 (3.4)	0 (0)	1 (0.2)	
All of the above.	66 (62.8)	19 (50)	13 (65)	60 (40)	19 (65.5)	33 (47.1)	210 (51)	
Do not know.	8 (7.6)	9 (23.7)	2 (20)	30 (20)	5 (17.2)	21 (30)	75 (18.2)	
**A serious ADR must be reported to the national PV center in?**								0.384
7 days	28 (26.7)	4 (10.5)	6 (30)	42 (28)	13 (44.8)	9 (12.9)	102 (24.8)	
10 days	6 (5.7)	2 (5.3)	1 (5)	10 (6.7)	1 (3.4)	5 (7.1)	25 (6.1)	
14 days	2 (1.9)	2 (5.3)	0 (0)	2 (1.3)	0 (0)	2 (2.6)	8 (1.9)	
15 days	13 (12.4)	5 (13.1)	5 (25)	25 (16.7)	6 (20.7)	14 (20)	68 (16.5)	
1 month	1 (0.9)	1 (2.6)	0 (0)	2 (1.3)	0 (0)	1 (1.4)	5 (1.2)	
Don’t know	55 (52.4)	24 (63.1)	8 (40)	69 (46)	9 (31)	39 (55.7)	204 (49.5)	
**ADR-related patients encountered during work experience?**								0.001
None	31 (29.5)	24 (63.1)	9 (45)	88 (58.7)	14 (48.3)	37 (52.8)	203 (49.3)	
1–5	42 (40)	10 (26.3)	11 (55)	36 (24)	12 (41.4)	6 (8.6)	117 (28.4)	
6–10	14 (13.3)	1 (2.6)	0 (0)	12 (8)	1 (3.4)	10 (14.3)	38 (9.2)	
11–15	1 (0.9)	0 (0)	0 (0)	3 (2)	2 (6.9)	9 (12.9)	15 (3.6)	
16–20	1 (0.9)	0 (0)	0 (0)	1 (0.7)	0 (0)	2 (2.8)	4 (1)	
More than 20	16 (15.2)	3 (7.9)	0 (0)	10 (6.7)	0 (0)	6 (8.6)	35 (10)	

*Explaining only one response was allowed,

**P-value: Chi square test, PV: Pharmacovigilance, ADR: Adverse drug reaction.

### Source of information and responsible person for PV and ADR reporting (multiple choice questions: More than one response was allowed)

Internet (n = 294; 71.4%) and drug package inserts/leaflets (n = 195; 47.3%) were the main sources of information utilized by HCPs about PV and ADR. The majority of the paramedics (85.7%), medical doctors (72.4%), and nurses (79.7%) utilized the internet as a main source of information about ADR as compared to midwives (65.5%), pharmacists (65%), and dentists (52.6%). In terms of questions related to the responsible person for ADR reporting, most of the participants indicated a medical doctor/physician (47.6%; n = 196), pharmacist (40.8%; n = 168), and all HCPs (37.6%; n = 155). Moreover, the majority of the pharmacists (50%), paramedics (45.7%), and medical doctors (41.9%) respond with the correct answer that all HCPs are responsible for ADR reporting. A statistically significant difference was also measured (p-value < 0.05) (Table 4).

**Table 4:** Source of information and responsible person for PV and ADR reporting (n = 412).

*Multiple choice questions	Responses	Doctor (n = 105)	Dentist (n = 38)	Pharmacist (n = 20)	Nurse (n = 150)	Midwife (n = 29)	Paramedic (n = 70)	Total	**P-value
**Source of information about PV and ADR?** *									
Internet	Yes	76 (72.4)	20 (52.6)	13 (65)	106 (70.7)	19 (65.5)	60 (85.7)	294 (71.4)	0.012
	No	29 (27.6)	18 (47.4)	7 (35)	44 (29.3)	10 (34.5)	10 (14.3)	118 (28.6)	
Scientific journal articles	Yes	33 (31.4)	9 (23.7)	9 (45)	20 (13.3)	4 (13.8)	20 (28.6)	95 (23.1)	0.001
	No	72 (68.6)	29 (76.3)	11 (55)	130 (86.7)	25 (86.2)	50 (71.4)	317 (76.9)	
Classical textbooks	Yes	23 (21.9)	5 (13.2)	5 (25)	11 (7.3)	1 (3.4)	12 (17.1)	57 (13.8)	0.006
	No	82 (78.1)	33 (86.8)	15 (75)	139 (92.7)	28 (96.6)	58 (82.9)	355 (86.2)	
Package inserts	Yes	56 (53.3)	9 (23.7)	12 (60)	60 (40)	15 (51.7)	43 (61.4)	195 (47.3)	0.001
	No	49 (46.7)	29 (76.3)	8 (40)	90 (60)	14 (48.3)	27 (38.6)	217 (52.7)	
Advertisement brochures	Yes	3 (2.9)	1 (2.6)	2 (10)	2 (1.3)	2 (6.9)	1 (1.4)	11 (2.7)	0.180
	No	102 (97.1)	37 (97.4)	18 (90)	148 (98.7)	27 (93.1)	69 (98.6)	401 (97.3)	
PV contact person	Yes	32 (30.5)	10 (26.3)	13 (65)	68 (45.3)	19 (65.5)	28 (40)	170 (41.3)	0.001
	No	73 (69.5)	28 (73.7)	7 (35)	82 (54.7)	10 (34.5)	42 (60)	242 (58.7)	
Pharmaceutical representative	Yes	12 (11.4)	2 (5.3)	4 (20)	5 (3.3)	9 (31)	2 (2.8)	34 (8.3)	0.001
	No	93 (88.6)	36 (94.7)	16 (80)	145 (96.7)	20 (69)	68 (97.1)	378 (91.7)	
**Responsible person for PV and ADR reporting?** *									
Doctor/physician	Yes	57 (54.3)	14 (36.8)	13 (65)	68 (45.3)	20 (69)	24 (34.3)	196 (47.6)	0.005
	No	48 (45.7)	24 (63.2)	7 (35)	82 (54.7)	9 (31)	46 (65.7)	216 (52.4)	
Pharmacist	Yes	38 (36.2)	17 (44.7)	10 (50)	74 (49.3)	19 (65.5)	10 (14.3)	168 (40.8)	0.001
	No	67 (63.8)	21 (55.3)	10 (50)	76 (50.7)	10 (34.5)	60 (85.7)	244 (59.2)	
Nurse	Yes	24 (22.9)	2 (5.3)	7 (35)	51 (34)	11 (37.9)	8 (11.4)	103 (25)	0.001
	No	81 (77.1)	36 (94.7)	13 (65)	99 (66)	18 (62.1)	62 (88.6)	309 (75)	
Dentist	Yes	25 (23.8)	10 (26.3)	7 (35)	14 (9.3)	3 (10.3)	6 (8.6)	65 (15.8)	0.001
	No	80 (76.2)	28 (73.7)	13 (65)	136 (90.7)	26 (89.7)	64 (91.4)	347 (84.2)	
Midwife	Yes	3 (2.9)	0 (0)	4 (20)	15 (10)	7 (24.1)	3 (4.3)	32 (7.8)	0.001
	No	102 (97.2)	38 (100)	16 (80)	135 (90)	22 (75.9)	67 (95.7)	380 (92.2)	
Paramedic	Yes	3 (2.9)	2 (5.3)	1 (5)	13 (8.7)	1 (3.4)	5 (7.1)	25 (6.1)	0.514
	No	102 (97.2)	36 (94.7)	19 (95)	137 (91.3)	28 (96.6)	65 (92.9)	387 (93.9)	
All HCPs	Yes	44 (41.9)	7 (14.4)	10 (50)	50 (33.3)	12 (41.4)	32 (45.7)	155 (37.6)	0.044
	No	61 (58.1)	31 (81.6)	10 (50)	100 (66.7)	17 (58.6)	38 (54.3)	257 (62.4)	
None of the above	Yes	0 (0)	0 (0)	0 (0)	0 (0)	1 (3.4)	0 (0)	1 (0.2)	0.02
	No	105 (100)	38 (100)	20 (100)	150 (100)	28 (96.6)	70 (100)	411 (99.8)	
Don’t know	Yes	8 (7.6)	8 (21)	1 (5)	15 (10)	1 (3.4)	18 (25.7)	51 (12.4)	0.001
	No	97 (92.4)	30 (79)	19 (95)	135 (90)	28 (96.6)	52 (74.3)	361 (87.6)	

*Explaining that more than one response was allowed,

**P-value: Chi square test, PV: Pharmacovigilance; ADR: Adverse drug reaction.

### Attitude toward PV and ADRs among HCPs

A high proportion of the HCPs agreed/strongly agreed that documentation of ADR is important (85.7% n = 353) for a better healthcare system. Of these most of the pharmacists (80%), midwives (62.1%), and medical doctors strongly agreed with this statement as compared to other HCPs groups. Most of the HCPs strongly indicated that ADR (serious or non-serious) should be reported (81.5%; n = 336), reporting of ADR makes a significant contribution to patient safety (86.6%; n = 357) and ADR reporting should be made compulsories for all HCPs (71.3%; 294). In these statements’ pharmacists, midwives, and medical doctors strongly agreed as compared to other HCPs groups. The majority of the participants (78.1%; n = 322) agreed/strongly agreed that PV should be included as a main topic in medical education. A higher percentage of HCPs including pharmacists (70%), medical doctors (60.9%), midwives (58.6%), paramedics (58.6%), nurses (48%), and dentists (28.9%) strongly agreed with this statement. Moreover, the majority of the HCPs (86.2%; n = 355) agreed/strongly agreed that HCPs who are trained in the field of PV can play a better role in ADR reporting. Concerning the profession, pharmacists (75%), medical doctors (66.7%), midwives (51.7), paramedics (57.1%), nurses (51.3%), and dentists (42.1%) strongly agreed to this statement. Moreover, a higher proportion of HCPs (70% pharmacists, 58.6% midwives, 53.3% medical doctors, 51.4% paramedics, 49.3% nurses, and 34.2% dentists) were strongly agreed that all HCPs need education about PV and ADR reporting systems. A statistically significant difference was also observed between professions for most of the attitude-related variables (p-value < 0.05). Complete details are given in Table 5. The mean attitude score was 38.37 ±7.305 (range 9–45), with 71.1% (n = 293) HCPs had positive attitudes, 25% (n = 103) had moderate and 3.9% (n = 16) had negative attitude levels regarding PV and ADRs. Pharmacists, medical doctors, and midwives had a more positive attitude as compared to other HCPs. A statistically significant difference was also observed for HCPs’ professions and attitude scores (p = value 0.000) (S1 Table).

**Table 5:** Attitude-related questions and profession comparison (n = 412).

Questions	Doctor (n = 105)	Dentist (n = 38)	Pharmacist (n = 20)	Nurse (n = 150)	Midwife (n = 29)	Paramedic (n = 70)	Total	*P-value
Documentation of ADR is important for a better healthcare system.								0.010
SD	2 (1.9)	1 (2.6)	0 (0)	9 (6)	1 (3.4)	2 (2.8)	15 (3.6)	
D	0 (0)	0 (0)	0 (0)	2 (1.3)	0 (0)	2 (2.8)	4 (1)	
U	7 (6.7)	6 (15.8)	0 (0)	9 (6)	3 (10.3)	15 (21.4)	40 (9.7)	
A	37 (35.2)	14 (36.8)	4 (20)	64 (42.7)	7 (24.1)	20 (28.6)	146 (35.4)	
SA	59 (56.2)	17 (44.7)	16 (80)	66 (44)	18 (62.1)	31 (44.3)	207 (50.2)	
Any ADR (serious or non-serious) should be reported spontaneously.								0.021
SD	2 (1.9)	0 (0)	1 (5)	6 (4)	1 (3.4)	3 (4.3)	13 (3.2)	
D	1 (0.9)	2 (5.3)	0 (0)	4 (2.7)	1 (3.4)	2 (2.8)	10 (2.4)	
U	10 (9.5)	6 (15.8)	0 (0)	16 (10.7)	2 (6.9)	19 (27.1)	53 (12.9)	
A	40 (38.1)	18 (47.4)	5 (25)	61 (40.7)	8 (27.6)	16 (22.8)	148 (35.9)	
SA	52 (49.5)	12 (31.6)	14 (70)	63 (42)	17 (58.6)	30 (42.8)	188 (45.6)	
Reporting ADR makes a significant contribution to patient safety.								0.014
SD	0 (0)	0 (0)	0 (0)	5 (3.3)	1 (3.4)	3 (4.3)	9 (2.2)	
D	2 (1.9)	0 (0)	0 (0)	2 (1.3)	0 (0)	0 (0)	4 (1)	
U	7 (6.7)	7 (18.4)	0 (0)	12 (8)	3 (10.3)	13 (18.5)	42 (10.2)	
A	29 (27.6)	15 (39.5)	2 (10)	52 (34.7)	5 (17.2)	14 (20)	117 (28.4)	
SA	67 (63.8)	16 (42.1)	18 (90)	79 (52.7)	20 (69)	40 (57.1)	240 (58.3)	
Reporting ADR should be made compulsories for all HCPs.								0.022
SD	2 (1.9)	1 (2.6)	0 (0)	10 (6.7)	1 (3.4)	4 (5.7)	18 (4.4)	
D	8 (7.6)	3 (7.9)	0 (0)	6 (4)	2 (6.9)	3 (4.3)	22 (5.3)	
U	17 (16.2)	11 (28.9)	0 (0)	27 (18)	3 (10.3)	20 (28.5)	78 (18.9)	
A	26 (24.8)	13 (34.2)	3 (15)	40 (26.7)	7 (24.1)	10 (14.3)	99 (24)	
SA	52 (49.5)	10 (26.3)	17 (85)	67 (44.7)	16 (55.2)	33 (47.1)	195 (47.3)	
PV should be included as a core topic in medical education.								0.058
SD	3 (2.9)	1 (2.6)	0 (0)	8 (5.3)	1 (3.4)	2 (2.8)	15 (3.6)	
D	3 (2.9)	3 (7.9)	0 (0)	5 (3.3)	0 (0)	1 (1.4)	12 (2.9)	
U	15 (14.3)	5 (13.1)	0 (0)	27 (18)	3 (10.3)	13 (18.6)	63 (15.3)	
A	20 (19)	18 (47.4)	6 (30)	38 (25.3)	8 (27.6)	13 (18.6)	103 (25)	
SA	64 (60.9)	11 (28.9)	14 (70)	72 (48)	17 (58.6)	41 (58.6)	219 (53.2)	
ADR reporting notification is important for the healthcare system.								0.015
SD	3 (2.9)	0 (0)	0 (0)	5 (3.3)	1 (3.4)	2 (2.8)	11 (2.7)	
D	2 (1.9)	3 (7.9)	0 (0)	3 (2)	0 (0)	0 (0)	8 (1.9)	
U	5 (4.8)	8 (21)	0 (0)	13 (8.7)	2 (6.9)	13 (18.6)	41 (10)	
A	23 (21.9)	13 (34.2)	4 (20)	45 (30)	8 (24.1)	17 (24.3)	110 (26.7)	
SA	72 (68.6)	14 (36.8)	16 (80)	84 (56)	18 (62.1)	38 (54.3)	242 (58.7)	
Do you think that HCPs who are trained in the field of PV can play a better role in ADR reporting?								
SD	1 (0.9)	0 (0)	0 (0)	6 (4)	1 (3.4)	2 (2.8)	10 (2.4)	0.113
D	1 (0.9)	2 (5.3)	0 (0)	1 (0.7)	0 (0)	1 (1.4)	5 (1.2)	
U	8 (7.6)	7 (18.4)	0 (0)	13 (8.7)	3 (10.3)	11 (15.7)	42 (10.2)	
A	25 (23.8)	13 (34.2)	5 (25)	53 (35.3)	10 (34.5)	16 (22.9)	122 (29.6)	
SA	70 (66.7)	16 (42.1)	15 (75)	77 (51.3)	15 (51.7)	40 (57.1)	233 (56.6)	
Do you believe that all HCPs need education about PV and ADR reporting systems?								0.147
SD	1 (0.9)	0 (0)	0 (0)	4 (2.7)	1 (3.4)	3 (4.3)	9 (2.2)	
D	4 (3.8)	4 (10.5)	0 (0)	5 (3.3)	0 (0)	0 (0)	13 (3.2)	
U	20 (19)	10 (26.3)	1 (5)	20 (13.3)	3 (10.3)	14 (20)	68 (16.5)	
A	24 (22.9)	11 (28.9)	5 (25)	47 (31.3)	8 (27.6)	17 (24.3)	112 (27.2)	
SA	56 (53.3)	13 (34.2)	14 (70)	74 (49.3)	17 (58.6)	36 (51.4)	210 (51)	
Do you think that ADRs can even result in death?								0.002
SD	3 (2.9)	0 (0)	0 (0)	5 (3.3)	1 (3.4)	2 (2.8)	11 (2.7)	
D	2 (1.9)	4 (10.5)	0 (0)	1 (0.7)	0 (0)	2 (2.8)	9 (2.2)	
U	7 (6.7)	7 (18.4)	1 (5)	17 (11.3)	5 (17.2)	16 (22.9)	53 (12.9)	
A	20 (19)	15 (39.4)	8 (40)	41 (27.3)	10 (34.5)	16 (22.9)	110 (26.7)	
SA	73 (69.5)	12 (31.6)	11 (55)	86 (57.3)	13 (44.8)	34 (48.6)	229 (55.6)	

*P-value: Chi square test, SD: Strongly disagree, D: Disagree, U: Uncertain, A: Agree, SA: Strongly agree, PV: Pharmacovigilance, ADR: Adverse drug reaction; HCPs: Healthcare professionals.

### Practices of HCPs towards PV and ADRs

The findings show that more than sixty percent (62.4%; n = 257) of the HCPs didn’t notify or mentioned the ADR encountered on the patient’s clinical record. Among these, a considerable proportion of medical doctors (45.7%) and midwives (41.4%) claimed that they notified/mentioned the encountered ADR as compared to other HCPs groups. Only 32.5% (n = 134) kept the records of ADR and more than half of pharmacists (55%) and midwives (51.7%) indicated that they kept the records of ADRs as compared to the lower percentage of nurses (41.3%), medical doctors (23.8%), paramedics (21.4%) and dentists (15.8%). Only 13.1% (n = 54) of the participants had ever reported ADR to a hospital/national PV center with a higher proportion among pharmacists (55%) as compared to nurses (15.3%), medical doctors (10.5%), midwives (10.3%) and paramedics (8.6%). Moreover, all dentists included in the current study also claimed that they did not send a suspected ADR report. More than half (51.5%; n = 212) of the respondents reported that ADR reporting forms are not easily available in a healthcare institution. The proportion was 81.4%, 61.9%, 55.3%, 36.7%, 31%, and 25% among paramedics, medical doctors, dentists, nurses, midwives, and pharmacists, respectively. Most of the participants, including nurses (41.3%), midwives (34.5%), dentists (31.6%), and medical doctors (29.5%) claimed that they always read the package inserts of the medicine before giving it to patients as compared to paramedics (24.3%) and pharmacists (20%). About 62.9% (n = 259) of the participants indicated that they always counsel patients about the side effects and possible ADRs of drugs and a higher proportion was observed among midwives (82.8%), pharmacists (70%), paramedics (68.6%), and doctors (61.9%). A statistically significant difference (p-value < 0.05) between the profession of HCPs for most of the practice-related questions **(**Table 6**)**. Moreover, the overall mean practice score was 3.15±2.141 (min-max = 0–8). The majority of the HCPs (n = 381; 92.5%) had poor practices towards PV and ADRs. Of these, the proportion of paramedics, dentists, medical doctors, nurses, midwives, and pharmacists was 98.6%, 97.4%, 96.2%, 89.3%, 82.8%, and 80%, respectively. Moreover, only 7.1% (n = 31) of the HCPs had good practices, and pharmacists, midwives and nurses had more good practices than other HCPs group. A statistically significant difference was also observed for HCPs professions and practice scores (p = 0.002) (S1 Table).

**Table 6:** Practice-related questions and profession comparison (n = 412).

Questions	Responses	Doctor (n = 105)	Dentist (n = 38)	Pharmacist (n = 20)	Nurse (n = 150)	Midwife (n = 29)	Paramedic (n = 70)	Total	*P-value
Notified/mentioned the ADR encountered on the patient’s clinical record.									0.116
	No	57 (54.3)	30 (79)	13 (65)	92 (61.3)	17 (58.6)	48 (68.6)	257 (62.4)	
	Yes	48 (45.7)	8 (21)	7 (35)	58 (38.7)	12 (41.4)	22 (31.4)	155 (37.6)	
Keep records of ADR.									0.001
	No	80 (76.2)	32 (84.2)	9 (45)	88 (58.7)	14 (48.3)	55 (78.6)	278 (67.5)	
	Yes	25 (23.8)	6 (15.8)	11 (55)	62 (41.3)	15 (51.7)	15 (21.4)	134 (32.5)	
Ever sent a suspected ADR report to a hospital/national PV center.									0.001
	No	94 (89.5)	38 (100)	9 (45)	127 (84.7)	26 (89.7)	64 (91.4)	358 (86.9)	
	Yes	11 (10.5)	0 (0)	11 (55)	23 (15.3)	3 (10.3)	6 (8.6)	54 (13.1)	
Hospital’s HCPs trained in how to report ADR.									0.001
	No	85 (81)	26 (68.4)	7 (35)	78 (52)	12 (41.4)	64 (91.4)	272 (66)	
	Yes	20 (19)	12 (31.6)	13 (65)	72 (48)	17 (58.6)	6 (8.6)	140 (34)	
ADR reporting forms are easily accessible in a healthcare institution.									0.001
	No	65 (61.9)	21 (55.3)	5 (25)	55 (36.7)	9 (31)	57 (81.4)	212 (51.5)	
	Yes	40 (38.1)	17 (44.7)	15 (75)	95 (63.3)	20 (69)	13 (18.6)	200 (48.5)	
Always read the package inserts of the medicine before giving to patients.									0.98
	No	74 (70.5)	26 (68.4)	16 (80)	88 (58.7)	19 (65.5)	53 (75.7)	276 (67)	
	Yes	31 (29.5)	12 (31.6)	4 (20)	62 (41.3)	10 (34.5)	17 (24.3)	136 (33)	
Advise patients to read the drug leaflets every time									0.001
	No	58 (55.2)	25 (65.8)	12 (60)	70 (46.7)	11 (37.9)	17 (24.3)	193 (46.8)	
	Yes	47 (44.8)	13 (34.2)	8 (40)	80 (53.3)	18 (62.1)	53 (75.7)	219 (53.2)	
Always counsel patients about the side effects and possible ADRs of drugs.									0.55
	No	40 (39)	20 (52.6)	6 (30)	60 (40)	5 (17.2)	22 (31.4)	153 (37.1)	
	Yes	65 (61.9)	18 (47.4)	14 (70)	90 (60)	24 (82.8)	48 (68.6)	259 (62.9)	

*P-value: Chi square test, PV: Pharmacovigilance, ADR: Adverse drug reaction; HCPs: Healthcare professionals.

### Factors associated with poor ADR reporting practice

A univariable logistic regression showed that the HCPs’ profession, district and lack of training were the predictors of poor ADR reporting practice. The findings of the multivariate logistic regression also measured that pharmacist and lack of prior PV training (p-value < 0.05) were linked with the poor practice of ADR reporting **(Table 7)**.

**Table 7:** Logistic regression (Univariable and multivariable) analysis of ADR reporting practice.

Variables	ADR reporting (Yes: 54 and No: 358)		Adjusted OR	Lower and upper 95% CI	P-value
	Yes, n(%)	No, n(%)			
**Gender**					0.887*
Female	34 (63)	229 (64)	1	-	-
Male	20 (37)	129 (36)	0.731	0.346–1.547	0.413**
**Profession**					0.000*
Doctor	11 (20.4)	94 (26.2)	1	-	-
Dentist	0 (0)	38 (10.6)	0.000	0.000–0.000	0.997**
Pharmacist	11 (20.4)	9 (2.5)	7.090	1.895–26.535	0.004**
Nurse	23 (42.6)	127 (35.5)	1.205	0.477–3.047	0.693**
Midwife	3 (5.5)	26 (7.2)	0.460	0.096–2.198	0.331**
Paramedic	6 (11.11)	64 (17.9)	0.624	0.076–5.139	0.661**
**Workplace**					0.314*
Family healthcare center	0 (0)	10 (2.8)	1	-	
Government/Public hospital	40 (74)	213 (59.5)	119695659.135	0.000–0.000	0.999**
Integrated district hospital	1 (1.8)	12 (3.3)	56653835.595	0.000–0.000	0.999*
University hospital	2 (3.7)	30 (8.4)	134791441.972	0.000–0.000	0.999**
Private hospital	4 (7.4)	23 (6.4)	113280405.737	0.000–0.000	0.999**
112 Control command center	7 (12.9)	70 (19.5)	345892821.806	0.000–0.000	0.999**
**District**					0.000*
Cukurova	17 (31.5)	48 (13.4)	1	-	-
Seyhan	2 (3.7)	34 (9.5)	0.259	0.039–1.730	0.163**
Yuregir	7 (12.9)	110 (30.7)	0.230	0.068–0.783	0.019**
Saricam	1 (1.8)	17 (4.7)	0.190	0.017–2.108	0.176**
Ceyhan	0 (0)	19 (5.3)	0.000	0.000–0.000	0.998**
Kozan	17 (31.5)	75 (20.9)	0.514	0.179–1.480	0.218**
Imamoglu	4 (7.4)	7 (1.9)	1.630	0.305–8.716	0.568**
Karaisali	6 (11.1)	37 (10.3)	0.339	0.094–1.224	0.099**
Kararatas	0 (0)	5 (1.4)	0.000	0.000–0.000	0.999**
Yumurtalik	0 (0)	6 (1.7)	0.000	0.000–0.000	0.999**
**Work experience**					0.888*
1–5 years	10 (18.5)	76 (21.2)	1	-	-
6–10 years	11(20.4)	76 (21.2)	1.226	0.399–3.765	0.722**
11–15 years	12 (22.2)	86 (24.1)	0.890	0.304–2.607	0.832**
More than 15	21 (38.9)	120 (33.5)	1.033	0.383–2.790	0.949**
**PV training**					0.000*
Yes	40 (74)	123 (34.4)	1	-	-
No	14 (26)	235 (65.6)	0.172	0.076–0.390	0.000**

*Univariable regression,

** Multivariable regression, PV: Pharmacovigilance, ADR: Adverse drug reaction.

### HCPs’ barriers to PV and ADRs reporting

The main barriers which were supposed to discourage ADR reporting by the HCPs were higher workload (n = 263; 63.8%), thinking that a single ADR report makes no impact (n = 262; 63.6%), lack of a professional atmosphere to discuss ADR (n = 214; 51.9%), insufficient financial support by health care authorities (n = 214; 51.9%) and lack of knowledge (n = 185; 44.9%). Similarly, with respect to the profession, a higher proportion of medical doctors (74.3%), nurses (63.3%), and midwives (62.1%) reported that a higher workload is the main discouraging factor for ADR reporting as compared to other HCPs. Thinking that a single ADR report makes no impact was the second main barrier as per HCPs’ views and most frequently pharmacists (90%) followed by midwives (65.5%) and medical doctors (64.8%) were agreed with this statement. Paramedics (57.1%), medical doctors (56.2%) and nurses (50.7%), and pharmacists (50%) highlighted that the lack of a professional atmosphere to discuss ADR is also a leading factor. Insufficient financial support by health care authorities was prevalent among medical doctors (65.7%) followed by paramedics (51.4%), nurses (48%), and pharmacists (45%). Lack of knowledge regarding the detection of ADRs was more commonly indicated by pharmacists (70%), midwives (51.7%), and medical doctors (48.6%). All HCPs reported various factors as per their point of view. A statistically significant difference was also reported (p-value < 0.05). A details comparison between barriers and the HCPs profession is given in Table 8.

**Table 8:** Barriers-related questions and profession comparison (n = 412).

Barriers	Responses	Doctor (n = 105)	Dentist (n = 38)	Pharmacist (n = 20)	Nurse (n = 150)	Midwife (n = 29)	Paramedic (n = 70)	Total	*P-value
Non-existence of a PV reporting center in the hospital.									0.001
	Yes	39 (37.1)	14 (36.8)	12 (60)	87 (58)	17 (58.6)	9 (12.8)	178 (43.2)	
	No	11 (10.5)	3 (7.9)	2 (10)	22 (14.7)	1 (3.4)	24 (34.3)	63 (15.3)	
	I don’t know	55 (52.4)	21 (55.3)	6 (30)	41 (27.3)	11 (37.9)	37 (52.9	171 (41.5)	
Lack of training/educational support.									0.001
	Yes	19 (18.1)	8 (21)	2 (10)	55 (36.7)	17 (58.6)	3 (4.3)	104 (25.2)	
	No	27 (25.7)	7 (18.4)	7 (35)	22 (14.7)	3 (10.3)	18 (25.7)	84 (20.4)	
	I don’t know	59 (56.2)	23 (60.5)	11 (55)	73 (48.6)	9 (31)	49 (70)	224 (54.4)	
Unavailability of ADR reporting forms in a health care setting.									0.001
	Yes	37 (35.2)	7 (18.4)	11 (55)	85 (56.7)	20 (69)	12 (17.1)	172 (41.7)	
	No	27 (25.7)	10 (26.3)	5 (25)	27 (18)	2 (6.9)	27 (38.6)	98 (23.8)	
	I don’t know	41 (39.1)	21 (55.3)	4 (20)	38 (25.3)	7 (24.1)	31 (44.3)	142 (34.5)	
More time-consuming									0.001
	Yes	42 (40)	8 (21)	3 (15)	50 (33.3)	8 (27.6)	24 (34.3)	135 (32.8)	
	No	41 (39)	18 (47.4)	17 (85)	68 (45.3)	17 (58.6)	18 (25.7)	179 (43.4)	
	I don’t know	22 (21)	12 (31.6)	0 (0)	32 (21.3)	4 (13.8)	28 (40)	98 (23.8)	
Fear to harm the confidence of patients.									0.020
	Yes	16 (15.2)	1 (2.6)	2 (10)	30 (20)	4 (13.8)	8 (11.4)	61 (14.8)	
	No	71 (67.7)	24 (63.2)	17 (85)	91 (60.7)	22 (75.9)	41 (58.6)	266 (64.6)	
	I don’t know	18 (17.1)	13 (34.2)	1 (5)	29 (19.3)	3 (10.3)	21 (30)	85 (20.6)	
Lack of knowledge									0.007
	Yes	51 (48.6)	13 (34.2)	14 (70)	68 (45.3)	15 (51.7)	24 (34.3)	185 (44.9) **	
	No	34 (32.4)	11 (28.9)	4 (20)	53 (35.3)	11 (37.9)	19 (27.1)	132 (32)	
	I don’t know	20 (19)	14 (36.8)	2 (10)	29 (19.3)	3 (10.3)	27 (38.6)	95 (23.1)	
Reporting forms are too complicated.									0.001
	Yes	24 (22.9)	7 (18.4)	4 (20)	46 (30.7)	7 (24.1)	8 (11.4)	96 (23.3)	
	No	30 (28.6)	11 (28.9)	14 (70)	58 (38.7)	15 (51.7)	24 (34.3)	152 (36.9)	
	I don’t know	51 (48.5)	20 (52.6)	2 (10)	46 (30.6)	7 (24.1)	38 (54.3)	164 (39.8)	
Fear of legal liability									0.005
	Yes	28 (26.7)	2 (5.3)	4 (20)	46 (30.6)	8 (27.6)	16 (22.8)	104 (25.2)	
	No	58 (55.2)	19 (50)	13 (65)	69 (46)	17 (58.6)	29 (41.4)	205 (49.8)	
	I don’t know	19 (18.1)	17 (44.7)	3 (15)	35 (23.3)	4 (13.8)	25 (35.7)	103 (25)	
Lack of motivation									0.093
	Yes	44 (41.9)	6 (15.8)	5 (25)	46 (30.6)	10 (34.5)	22 (31.4)	133 (32.3)	
	No	47 (44.8)	23 (60.5)	12 (60)	70 (46.7)	15 (51.7)	28 (40)	195 (47.3)	
	I don’t know	14 (13.3)	9 (23.7)	3 (15)	34 (22.6)	4 (13.8)	20 (28.6)	84 (20.4)	
No idea how to report ADR									0.001
	Yes	57 (54.3)	12 (31.6)	3 (15)	53 (35.3)	9 (31)	33 (47.1)	167 (40.5)	
	No	32 (30.5)	13 (34.2)	14 (70)	60 (40)	18 (62.1)	14 (20)	151 (36.7)	
	I don’t know	16 (15.2)	13 (34.2)	3 (15)	37 (24.7)	2 (6.9)	23 (32.9)	94 (22.8)	
Inadequate knowledge of pharmacotherapy in detecting ADR.									0.004
	Yes	47 (44.8)	14 (36.8)	7 (35)	57 (38)	11 (37.9)	30 (42.8)	166 (40.3)	
	No	44 (41.9)	13 (34.2)	13 (65)	56 (37.3)	16 (55.2)	18 (25.7)	160 (38.8)	
	I don’t know	14 (13.3)	11 (28.9)	0 (0)	37 (24.7)	2 (6.9)	22 (31.4)	86 (20.9)	
Lack of a professional atmosphere to discuss ADR.									0.001
	Yes	59 (56.2)	17 (44.7)	10 (50)	76 (50.7)	12 (41.4)	40 (57.1)	214 (51.9) **	
	No	26 (24.8)	11 (28.9)	9 (45)	42 (28)	14 (48.3)	6 (8.6)	108 (26.2)	
	I don’t know	20 (19)	10 (26.3)	1 (5)	32 (21.3)	3 (10.3)	24 (34.3)	90 (21.8)	
Higher workload									0.040
	Yes	78 (74.3)	20 (52.6)	12 (60)	95 (63.3)	18 (62.1)	40 (57.1)	263 (63.8) **	
	No	15 (14.3)	11 (28.9)	6 (30)	33 (22)	9 (31)	11 (15.7)	85 (20.6)	
	I don’t know	12 (11.4)	7 (18.4)	2 (10)	22 (14.7)	2 (6.9)	19 (27.1)	64 (15.5)	
Insufficient financial support									0.001
	Yes	69 (65.7)	15 (39.5)	9 (45)	72 (48)	13 (44.8)	36 (51.4)	214 (51.9) **	
	No	21 (20)	12 (31.6)	8 (40)	52 (34.7)	11 (37.9)	8 (11.4)	112 (27.2)	
	I don’t know	15 (14.3)	11 (28.9)	3 (15)	26 (17.3)	5 (17.2)	26 (37.1)	86 (20.9)	
Forgetfulness is a barrier									0.53
	Yes	41 (39)	9 (23.7)	4 (20)	63 (42)	12 (41.4)	28 (40)	157 (38.1)	
	No	50 (47.6)	18 (47.4)	11 (55)	69 (46)	14 (48.3)	24 (34.3)	186 (45.1)	
	I don’t know	14 (13.3)	11 (28.9)	5 (25)	18 (12)	3 (10.3)	18 (25.7)	69 (16.7)	
Thinking that a single ADR report makes no impact									0.007
	Yes	68 (64.8)	23 (60.5)	18 (90)	94 (62.7)	19 (65.5)	40 (57.1)	262 (63.6) **	
	No	21 (20)	6 (15.8)	1 (5)	35 (23.3)	7 (24.1)	7 (10)	77 (18.7)	
	I don’t know	16 (15.2)	9 (23.7)	1 (5)	21 (14)	3 (10.3)	23 (32.9)	73 (17.7)	
Other coworkers are not reporting ADR cases.									0.102
	Yes	19 (18.1)	8 (21)	3 (15)	37 (24.7)	8 (27.6)	15 (21.4)	90 (21.8)	
	No	30 (28.6)	7 (18.4)	2 (10)	46 (30.7)	9 (31)	11 (15.7)	105 (25.5)	
	I don’t know	56 (53.3)	23 (60.5)	15 (75)	67 (44.6)	12 (41.4)	44 (62.9)	217 (52.7)	
Thinking that ADR reporting is not a duty.									0.004
	Yes	25 (23.8)	7 (18.4)	3 (15)	29 (19.3)	7 (24.1)	14 (20)	85 (20.6)	
	No	55 (52.4)	20 (52.6)	17 (85)	85 (56.7)	15 (51.7)	24 (34.3)	216 (52.4)	
	I don’t know	25 (23.8)	11 (28.9)	0 (0)	36 (24)	7 (24.1)	32 (45.7)	111 (26.9)	

*P-value: Chi square test, PV: Pharmacovigilance, ADR: Adverse drug reaction,

**Main barriers.

## Discussion

To the best of our knowledge, this is a Türkiye’s first study on PV and ADRs reporting among various HCPs working in different healthcare settings in 10 districts of Adana. The purpose of the current study was to evaluate HCPs’ KAPs regarding PV and ADR reporting, as well as to identify the major barriers and factors that prevent the implementation of a PV and ADR reporting system. Concerning knowledge scores, more than half of the HCPs (51.9%) had poor knowledge in our study. Similar findings were also reported in the Ethiopian study (58.3%) [15]. However, this value was lower in a study carried out in South-West Nigeria (21.7%) [37]. In the current study, 68.2% of the respondents indicated that they were aware of the terms “PV” and “ADR”. However, despite the awareness, most of the participants (65%) did not know about filling out the ADR reporting form. Comparable results were also observed in the Ethiopia study (63.2%) [15]. Additionally, our study showed that, only one-fourth (25%) and 51.9% of the HCPs were aware of the International and hospital PV and ADR monitoring center, respectively. Unawareness of international, national, and hospital-based PV centers among HCPs was also observed in previous studies conducted in various countries [15, 38–40]. These data highlighted the lack of knowledge about the international and local PV system components. Moreover, in the present study, 71.4% of the respondents correctly choose the definition of PV. Previously published studies conducted in Pakistan [41] and Nepal [42] also reported that 54.9% and 47.3% of the HCPs correctly select the PV definition. Similarly, 69.7% of respondents indicated the exact definition of ADRs. This value was higher as compared to studies carried out in Pakistan (62%) [43] and Ethiopia (29.3%) [15]. It is recommended to conduct periodic educational intervention to improve knowledge scores among HCPs [12]. Therefore, healthcare authorities should provide compulsory periodic PV-related courses and mandatory training for all HCPs.

In this study, a higher proportion of the participants (71.1%) had positive attitudes score and the attitude of pharmacists and medical doctors were quite encouraging as compared to other HCPs. A consistent finding was also reported in previous studies [17, 31, 37]. The positive attitude of respondents toward ADR reporting is an important factor because proper action can be taken to improve HCPs’ participation in ADR reporting by understanding their attitudes [15, 17]. The majority of the HCPs believed that documentation and reporting of ADRs are important for a better healthcare system and patient safety. These findings are consistent with studies conducted in Pakistan [31], South Africa [32], Nepal [44], and India [45]. Similarly, most of the HCPs considered that ADR reporting should be made compulsories for all HCPs. This finding was also reported by Nisa et al, [31] Alshammari et al, [39] and Ali et al [41]. The majority of the HCPs also believed that PV should be included as a main topic in medical education and all HCPs need education about PV and ADR reporting systems. Similar findings were also documented in prior studies [9, 15, 31, 37]. It is critical to raise awareness about ADR reporting, and education interventions have a positive impact on attitude and raising ADR reporting awareness among HCPs [12, 31]. A recent study also reported a significant improvements in attitude after an educational intervention [12]. Moreover, TÜFAM regulations mandate the inclusion of PV literature in educational program courses because there is no appropriate universal standard for PV training and teaching at the university level in Türkiye for students studying medicine, pharmacy, nursing, and other paramedical fields [10, 40, 46]. Therefore, periodic education and training are required to improve HCP attitudes and enhance PV activities.

As per the findings of current study, most of the HCPs had poor ADR reporting practice score. Although more than half of respondents (50.7%) encountered 1–5 to more than 20 ADRs during work experience, however, only 32.5% kept a record of an ADR and only 13.1% reported ADRs. Various studies have found similar trends, with the majority of HCPs failing to report any ADR despite encountering it during their practice [15, 17, 37, 47]. The rate of ADR reporting by various HCPs is very low in many countries [18, 32, 48, 49] including Türkiye [20–22, 50]. Our finding revealed that a higher proportion of pharmacists (55%) reported ADRs as compared to other HCPs. These findings were consistent with a previous study conducted in Thailand [38]. A higher value was reported in Saudi Arabia (71.3%) [51], Oman (69.2%) [52], Pakistan (67.6%) [17] and Ghana (66.7%) [53]. However, lower proportion of ADR reporting among pharmacist as compared to our findings was observed in Kuwait (26.8%) [54], Jordan (19.5%) [55] and Syria (10.8%) [56]. The provision of pharmaceutical care including PV activities is a professional responsibility for pharmacists [57]. Hospital pharmacists can not only identify and report ADRs, but also assist in reducing the financial burden associated with ADRs [17]. In Türkiye, like other HCPs, pharmacists are responsible for reporting ADRs to TÜFAM, and our study found that pharmacists are more aware of their responsibilities. However, more active involvement of hospital pharmacists is needed to ensure medicines safety by ADR reporting. Additionally, all dentists included in the current study also claimed that they did not send a suspected ADR report. These findings were consistent with previous studies [58–60]. Dental doctors prescribe a variety of therapeutic interventions, including allopathic medications such as local anesthetics, antibiotics, analgesics, and anti-inflammatory drugs [61]. Antibiotics and analgesics are two of the most common causes of ADRs [58, 61]. Oral signs and symptoms such as dry mouth, oral ulcers, taste changes, or swellings are reported to be caused by the medications’ adverse effects [60]. Therefore, the risk of ADRs in dental practice cannot be ignored and the active role of dentists is crucial for an effective PV system and patient safety [58, 61]. Despite the crucial role of the dentist in patient safety, PV is still the least understood and practiced in dentistry [62]. Similarly, only 8.6% of the paramedics claimed to report ADRs. No studies were found in a literature search specifically related to PV and ADR reporting among paramedics. It is also vital to urge the paramedics to report ADRs for the more effective PV programs because they have longer and closer relationships with patients [63]. Education and involvement of paramedics in PV and ADR reporting are highly recommended [63, 64]. It was also demonstrated that the number of reports of ADR increased by 148% shortly after educational interventions [65]. The findings of current study demand the immediate attention for PV training modules implementation as soon as possible and all HCPs should be made aware of the importance of ADR reporting. Therefore, strategies for periodic educational intervention among all HCPs are required to improve ADR reporting practice and patient care.

It is also important to mention that, all ADRs related to herbs, cosmetics, vaccines, new drugs, etc. must be reported. ADRs related to herbal and traditional medications [66, 67], cosmetics [34, 68], vaccines [69, 70], newly approved drugs [71], and unknown ADRs to old drugs [72, 73] are well recognized. However, only 51% of HCPs considered the mandatory reporting of all ADRs. Similar findings were also reported in the South African study [32]. There are various global databases for the spontaneous reporting of all ADRs such as WHO VigiBase [74], The United States Food and Drug Administration (FDA) Adverse Event Reporting System (AERS) [75], the Vaccine Adverse Event Reporting System (VAERS) [76], the European Medicines Agency (EMA) EudraVigilance (EV) [77]. TÜFAM utilizes a web-based approach (Vigiflow database) for the reporting of ADRs to the WHO–UMC database. TÜFAM developed an online ADR reporting form for ADR, adverse drug events, herbal drugs, vaccines [78], and cosmetics [79]. However, unawareness among HCPs regarding PV and ADR reporting methods is observed in the current study and also in previously published studies [37, 44]. There is a need for educating HCPs about PV, and ADR reporting structures and modalities for a better healthcare system.

In our study, more than half (60.4%) of the HCPs were untrained about PV and ADR reporting. Previous studies conducted in Nigeria (86.5%) [27], Saudi Arabia (76.3%) [80], South Africa (68.3%) [81], Ethiopia (55.5%) [15], Finland (48%) [48], and Ghana (37.1%) [53] reported that various proportion of HCPs had no training on PV and ADR reporting. Our results also showed that lack of training was the predictors of poor ADR reporting practice among HCPs. Previous studies demonstrated that education and training have a positive effect on HCPs’ willingness to report an ADR, awareness of PV, and increase ADR reporting rates [12, 32, 47]. It is also revealed that a lack of ADR reporting training was significantly associated with insufficient knowledge of PV and ADRs [12, 15]. Continuous education and training are very important for PV understanding and an increasing trend in ADR reporting was observed after training [47]. Additionally, a research study showed periodic training, lectures, education, regular newsletters on recent knowledge in the safety of the drug, information about new ADRs, and international safety information on drugs are the best ways to raise awareness about PV and the rate of ADR among HCPs [82]. In Türkiye, TÜFAM is primarily responsible for educating and training of HCPs. Additionally, supporting stakeholders including medical schools, pharmacology departments, and hospital PV centers are also accountable for PV-based awareness programs [10, 21]. However, TÜFAM and other stakeholders are unsuccessful to arrange training, education sessions, and reviews of HCPs’ actions in a timely and systematic way [9, 20]. Therefore, it is necessary to provide more training to HCPs on PV activities, ADR identification, the aim of ADR reporting, and the resources that are available for ADR reporting.

Our study identified several factors, including higher workload, the belief that a single ADR report has no impact, a lack of a professional atmosphere, a lack of support by health care authorities, and lack of knowledge as the main barriers that were intended to discourage reporting of ADRs among HCPs. Similarly, a study conducted in Pakistan observed the unavailability of a professional environment in the health setup to discuss an ADR and the lack of incentives for reporting ADRs as the main barrier among HCPs [41]. Another study carried out in Ethiopia revealed that poor knowledge and lack of training were predictors of poor ADR reporting practice [15]. Various studies highlighted many variables that may contribute to the under-reporting of ADRs, including a lack of time, ambiguity about the nature of drug reactions to report, not knowing where to report, and lack of ADR reporting forms and training [37, 83, 84]. Our recently published systematic review also highlighted that lack of time, uncertainty regarding ADRs and did not know where to report were the most frequently reported barriers to PV among HCPs in Türkiye [9]. A single-center local study reported that unawareness of the national PV system and spontaneous reporting of ADRs were the main discouraging factors [40]. It is highly suggested that improved activities of regional PV centers, interprofessional and collaborative links between HCPs, ongoing educational interventions (lectures, oral workshops, presentations, group discussions, and hands-on training), systematic follow-up by local health care policymakers, offering incentives to HCPs, implementation of mandatory reporting policy for HCPs and automation and utilization of artificial intelligence in the screening and reporting of ADRs are crucial for a better PV system in Türkiye and around the world [9, 11, 15, 82, 85].

### Limitation and strength

This research study had several limitations such as involving only one province and unequal distribution of respondents from the various HCPs groups. For example, there was low participation from pharmacists (4.8%), midwives (7%), and dentists (9.2%) out of the whole HCPs sample. We’re not sure why, but possible explanations include being hesitant to participate, too busy, or being on night duty at the 24-hour. However, nurses, doctors, and paramedics took part in more participation. Furthermore, qualitative research methodologies would have provided a more comprehensive understanding. Future study is needed to plan and address these matters. Convenience sampling was used to select the participants, so the sample may not accurately reflect the entire population. Another issue was the length of our survey (58 questions); some HCPs may be discouraged from participating solely because of this factor. The study was a cross-sectional study, so no causal inferences can be drawn. This study only included HCPs from the Adana region of Türkiye, which limits the generalizability of the findings. However, Adana is Türkiye’s fifth largest province (with a population of 1248988). Hence, it is anticipated that the results in the other areas would not differ significantly. The current study also relies on self-reported data, which can be subject to bias. Finally, our study identify the perceived barriers towards PV and ADR reporting but did not explore the specific reasons why HCPs had poor knowledge and practices, which could have provided insights for interventions to improve PV and ADR reporting. These points should be considered in future studies.

Regardless of these limitations, we believe that our findings are sound and will help authorities improve PV activities in the future. This is the first study in Türkiye on the KAP of HCPs (including paramedics) toward PV and ADR reporting and will provide baseline data for further research studies. We believe that the results would not change significantly and that limited knowledge of PV and ADR activities is a widespread national/global issue. The current study’s findings suggest that all HCPs should receive drug safety knowledge and training about ADR reporting. To accomplish this, a hands-on training and workshop system for dealing with ADR must be organized at the hospital level. Furthermore, cooperation amongst academic institutes, drug manufacturers, regulatory authorities, and HCPs should be expanded to sensitize reporting practices of ADRs.

## Conclusion

This study conclude that most of HCPs had poor knowledge and practice, but they had a positive attitude about PV and ADR reporting. Lack of training and under-reporting of ADRs was observed among HCPs. Barriers to under-reporting of ADR were also highlighted. Periodic training programs, educational interventions, systematic follow-up of HCPs by local healthcare authorities, interprofessional links between all HCPs, and implementation of mandatory reporting policy are crucial for better HCPs’ knowledge, practices, patient safety, and improved PV activities.

## Supporting information

S1 File Data collection tool (English and Turkish version questionnaires). (PDF)

S1 Table Knowledge, attitude and practice score level among various profession of HCP. (DOCX)
