# Supplementary material for: Evaluation of health care professionals’ knowledge, attitudes, practices and barriers to pharmacovigilance and adverse drug reaction reporting: A cross-sectional multicentral study
Source: PLoS One. 2023 May 24;18(5):e0285811. doi: 10.1371/journal.pone.0285811 (PMC10208525; doi:10.1371/journal.pone.0285811)
Supplement: S1 File — (PDF) [file pone.0285811.s001.pdf]

S1 File: Data collection tool (questionnaire)

EVALUATION OF HEALTH CARE PROFESSIONALS' KNOWLEDGE, ATTITUDES, PRACTICES AND BARRIERS TO PHARMACOVIGILANCE AND ADVERSE DRUG REACTION REPORTING: A CROSS-SECTIONAL MULTICENTRAL STUDY

The findings of the present study will be kept confidential and only be usedfor scientific research purposes. Personal identity information is not requested from the participants.

Serial no:_________________ Accept: ( ) Reject: ( ) Date: ……/……/…..

Section 1: Sociodemographic and general information

1. **Gender**: Female ☐ Male ☐
2. **Age** (years):____
3. **Profession:** a) Medical doctor b) Dentist c) Pharmacist d) Nurse e) Midwife f) Paramedic
4. **Workplace:** a) Family healthcare center b) Community healthcare center c) Government/Public hospital d) Integrated District hospital e) University Hospital f) Private hospital g) 112 Control Command Center
5. **District where you work:** a) Cukurova b) Seyhan c) Yuregir d) Saricam e) Ceyhan f) Kozan g) İmamoglu h) Karaisali i) Karatas j) Yumurtalik
6. **Working experience: a)** 1-5 years b) 6–10 years c) 11–15 years d) 15 or above
7. **Have you attended any training on pharmacovigilance (PV) before?:** a) Yes b) No

Section 2 (a). (Knowledge related questions).

Please indicate the extent with each statements to which you are agree or disagree.

Sr.no	Questions	Yes	No
Q1	Can you describe the PV term?	1	2
Q2	Can you define adverse drug reaction (ADR)?	1	2
Q3	There is any difference between ADRs and adverse events?	1	2
Q4	Have you ever read research publications and book on PV and ADRs?	1	2
Q5	Do you know how to fill the ADR reporting form?	1	2
Q6	Do you know about the international center for PV and ADR monitoring?	1	2
Q7	Could you explain the TÜFAM expansion?	1	2
Q8	Are you aware of the pharmacovigilance contact point of the hospital?	1	2

Section 2 (b): Please indicate the statement below to which you agree (Knowledge related questions).

1. **Which of the following is responsible for ADR reporting? (You can tick more than oneoption).**

a. Doctor/Physician b) Pharmacist c) Nurse d) Dentist
e. Midwife f) Paramedics g) All HCPs h) None of the
i. Do not know

2. **When did you first hear the term PV? (Tick only one option).**

a. In this survey
b. In training/continuing education programs
c. When I was a student
d. In congress/meetings
e. From the PV contact point of my institution
f. From a pharmaceutical company representative

3. **Which of the following definitions of PV is the most accurate? (Tick only one option).**

a. Activities of detecting, assessing, understanding & preventing adverse effects.
b. Detecting the type and incidence of ADRs after a drug is marketed.
c. The process of enhancing drug safety.
d. The science of monitoring ADRs in a hospital.
e. None of the above.
f. Do not know.

4. **Which of the following appropriately defines an ADR? (Tick only one option).**

a. Any undesirable effect of a medicine that occurs at normal doses during normal conditions ofuse.
b. Adverse health outcomes associated with irrational/ inappropriate use of drug.
c. Injury caused by the use of substandard/counterfeit medications.
d. Damage caused by a drug overdose.
e. None of the above.
f. Do not know.

5. **Which ADRs must be reported? (Tick only one option).**

a. All serious ADRs
b. ADRs to herbal drugs
c. ADRs to new drugs
d. ADRs to vaccines
e. ADRs due to the cosmetic use
f. Unknown ADRs to old drugs
g. All of the above
h. Do not know

6. **How many ADRs related patients have you encountered during your work experience?(Tick only one option).**

a. None b) 1-5 c) 6-10 d) 11-15 e) 16-20 f) More than 20

7. A serious ADR must be reported to the national PV center in? (Tick only one option).

a. 7 days b) 10 days c) 14 days d) 15 days e) 1 month f) Do not know

8. Where do you look up when you need information about an ADR? (You can tick morethan one option).

a. Search engines (internet) b) Scientific journal articles c) Classical textbooks

d. Package inserts e) Advertisement brochures/leaflets f) PV centre

g. Pharmaceutical company representative

Section 3: Please indicate the extent of response to each statement below (Attitude related questions).

Sr.no	Questions	SD	D	U	A	SA
Q1	Are documentation of ADR is important to thehealthcare system.	1	2	3	4	5
Q2	Any ADR (serious or non-serious) should be reportedspontaneously.	1	2	3	4	5
Q3	Reporting of ADR makes a significant contribution topatient safety.	1	2	3	4	5
Q4	Reporting ADR should be made compulsories for all HCPs.	1	2	3	4	5
Q5	PV should be included as a core topic in medicaleducation.	1	2	3	4	5
Q6	ADR reporting notification is important for thehealthcare system	1	2	3	4	5
Q7	Do you think that HCPs who are trained in the field ofPV can play a better role in ADR reporting.	1	2	3	4	5
Q8	Do you believe that all HCPs need education about PVand ADR reporting systems.	1	2	3	4	5
Q9	Do you think that ADRs can even result in death.	1	2	3	4	5

SD: Strongly disagree, D: Disagree, U: Uncertain, A: Agree, SA: Strongly agree.

Section 4: Please mark Yes (1) or No (2) for each question below (Practice related questions)

Sr.no	Questions	Yes	No
Q1	Have you mentioned/notified the ADR you encountered on the patient'sclinical record.	1	2
Q2	Do you keep records of ADR.	1	2
Q3	Have you ever sent a suspected ADR report to a hospital/national PVcenter.	1	2
Q4	Are your hospital's HCPs trained in how to report ADR?	1	2
Q5	Are ADR reporting forms easily accessible in your healthcareinstitution?	1	2
Q6	I always read the package inserts of the medicine before givingmedications to patients.	1	2
Q7	I advise patients to read the drug leaflets every time.	1	2
Q8	I always counsel patients about the side effects and possible ADRs ofdrugs.	1	2

Section 5: Please indicate the extent of response to each statement below (Barriers related questions).

Sr.no	Questions	Yes	No	I don't know
Q1	Non-existence of PV reporting center in the hospital.	1	2	3
Q2	Lack of the provision of training/educational support from PV centers to hospitals.	1	2	3
Q3	Unavailability of ADR reporting forms in your health care setting.	1	2	3
Q4	More time-consuming in ADR reporting.	1	2	3
Q5	Fear to harm the confidence of patients.	1	2	3
Q6	Insufficient knowledge in detecting ADRs.	1	2	3
Q7	ADR forms are too complicated.	1	2	3
Q8	I fear legal liability for the reported ADR.	1	2	3
Q9	I am not motivated to report ADR.	1	2	3
Q10	I have no idea how to report ADR.	1	2	3
Q11	Inadequate knowledge of pharmacotherapy in detectingADR.	1	2	3
Q12	Lack of a professional atmosphere to discuss ADR.	1	2	3
Q13	Higher workload on HCPs.	1	2	3
Q14	Insufficient financial support by health care authorities.	1	2	3
Q15	Forgetfulness is a barrier.	1	2	3
Q16	Thinking that a single ADR report makes no impact.	1	2	3
Q17	Other coworkers are not reporting ADR cases.	1	2	3
Q18	Thinking that ADR reporting is not a duty.	1	2	3

Thank you for your time and consideration

SAĞLIK SAĞLIK ÇALIŞANLARI PROFESYONELLERININ FARMAKOVIJILANS VE ADVERS ILAÇ REAKSIYONU BILDİRİMİ HAKKINDA BILGI, TUTUM, UYGULAMA VE ENGELLERININ DEĞERLENDIRILMESI: KESITSEL ÇOK MERKEZLI BIR ÇALIŞMA

Mevcut çalışmanın bulguları gizli tutulacak ve sadece bilimsel araştırma amacıyla kullanılacaktır. Çalışmaya katılanlardan kimlik bilgisi istenmemektedir.

Sıra No: ( ) Kabul ( ) Red Tarih:…/…./….

Bölüm 1: Sosyodemografik ve genel bilgiler

1. **Cinsiyet:** a) Kadın b) Erkek
2. **Yaş:……………………………………………..**
3. **Meslek:** a) Doktor b) Diş doktoru c) Eczacı d) Hemşire e) Ebe f) Paramedik
4. **Çalıştığınız kurum:** a) Aile Sağlık Merkezi (ASM) b) Toplum Sağlığı Merkezi (TSM)
   c) Kamu Hastanesi d) Entegre İlçe Hastanesi e) Üniversite hastanesi
   f) Özel Hastane g) 112 Kontrol Komuta Merkezi
5. **Çalıştığınız İlçe:** a) Çukurova b) Seyhan c) Yüreğir d) Sarıçam e) Ceyhan
   f) Kozan g) İmamoğlu h) Karaisalı ı) Karataş i) Yumurtalık
6. **Çalışma Deneyimi**: a) 1-5 yıl b) 6-10 yıl c) 11–15 yıl d) 15 veya üstü
7. **Daha önce farmakovijilans (FV) ile ilgili bir eğitime katıldınız mı?** a. Evet b. Hayır

Bölüm 2 (a): Lütfen aşağıdaki her bir ifadeye ne ölçüde katıldığınızı veya katılmadığınızı belirtiniz (Bilgi soruları).

No.	Sorular	Evet	Hayır
1	FV tanımını biliyormusunuz?	1	2
2	Advers ilaç reaksiyonlar (AIR'lar) tanımlayabilirmisiniz?	1	2
3	AIR'lar ile advers olaylar arasında herhangi bir fark var mı?	1	2
4	FV ve AIR ile ilgili araştırmaları ve kitaplarını hiç okudunuz mu?	1	2
5	AIR formunu nasıl dolduracağınızı biliyor musunuz?	1	2
6	Uluslararası FV ve AIR izleme merkezini biliyor musunuz?	1	2
7	TÜFAM’ın açılımını biliyormusunuz?	1	2
8	Hastanelerde FV irtibat noktasının olduğundan haberdar mısınız?	1	2

Bölüm 2 (b): Lütfen aşağıdaki kabul ettiğiniz cümleyi belirtiniz (Bilgi soruları).

1. AİR raporlamasından aşağıdakilerden hangisi/hangileri sorumludur (Birden fazlaseçenek işaretleyebilirsiniz)?

a. Doktor	b. Eczacı	c. Hemşire
d. Diş hekimi	e. Ebe	f. Paramedik
g. Tüm Sağlık çalışanları	h. Hiçbiri değil	i. Herhangi bir fikrim yok

2. FV terimini ilk ne zaman duydunuz (Tek seçeneği işaretleyiniz)?

a. Bu ankette
b. Eğitimlerde / sürekli eğitim programlarında
c. Öğrenciyken
d. Kongre / toplantılarda
e. Kurumumun FV irtibat noktasından
f. Bir ilaç firması temsilcisinden

3. Aşağıdakilerden hangisi FV'yi EN İYİ tanımlar (Tek seçeneği işaretleyiniz)?

a. Advers etkileri tespit etme, değerlendirme, anlama ve önleme bilimi ve faaliyetleri
b. Bir ilaç piyasaya sürüldükten sonra AIR'lerin tipini ve sıklığını tespit etme bilimi.
c. İlaçların güvenliğini artırma süreci.
d. Bir hastanede meydana gelen AIR'leri izleme bilimi
e. Yukarıdakilerin hiçbiri değil
f. Herhangi bir fikrim yok

4. Aşağıdakilerden hangisi bir AIR'yi doğru tanımlar (Tek seçeneği işaretleyiniz)?

a. Normal dozlarda meydana gelen bir ilacın zararlı veya istenmeyen etkileri.
b. Akılcı olmayan ilaç kullanımıyla ilişkili olumsuz sağlık sonuçları.
c. Standart altı / sahte ilaçların kullanımından kaynaklanan zarar.
d. Aşırı dozda ilaç kullanımında kaynaklanan zarar.
e. Yukarıdakilerin hiçbiri değil
f. Herhangi bir fikrim yok

5. Hangi AIR'ler rapor edilmelidir (Tek seçeneği işaretleyiniz)?

a. Tüm ciddi AIR'ler
b. Bitkisel ilaçlara ait AIR'ler
c. Yeni ilaçlara ait AIR'ler
d. Aşılara ait AIR'ler
e. Kozmetik ürünlere ait AIR'ler
f. Eski ilaçlara ait beklenmeyen AIR'ler
g. Yukarıdakilerin hepsi
h. Herhangi bir fikrim yok

6. İş tecrübeniz boyunca kaç tane AIR hastası ile karşılaştınız (Tek seçeneği işaretleyiniz)?

a) Hiçbiri b) 1-5 c) 6-10 d) 11-15 e) 16-20 f) 20'den fazla

7. Ulusal FV merkezine ciddi bir AIR kaç gün içerisinde bildirilmelidir (Tek seçeneğiişaretleyiniz)?

a. 7 b. 10 c. 14 d.15 e. 1 ay f. Herhangi bir fikrim yok

8. AIR hakkında bilgiye ihtiyaç duyduğunuzda hangi bilgi kaynaklarına bakarsınız (Birden fazla seçenek işaretleyebilirsiniz)?

a. İnternet b. Bilimsel dergi makaleleri c. Klasik ders kitapları d. İlaç prospektüsleri
e. Reklam broşürleri f. FV irtibat noktası g. İlaç firması temsilcisi

Bölüm 3. Lütfen aşağıdaki her bir ifadeye ne ölçüde katıldığınızı veya katılmadığınızı belirtin (Tutum soruları).

No	Sorular	KKM	KM	B	K	KK
1	Sağlık sistemi için AIR'nun dokümantasyonu önemlidir	1	2	3	4	5
2	Herhangi bir AIR (ciddi veya ciddi olmayan) spontanolarak raporlanmalı	1	2	3	4	5
3	AIR'nin raporlanması, hasta güvenliğine önemli bir katkı sağlıyor	1	2	3	4	5
4	Bir AIR'yi bildirmek tüm sağlık çalışanlarının mesleki bir zorunluluğudur	1	2	3	4	5
5	FV mezuniyet öncesi eğitime dahil edilmeli	1	2	3	4	5
6	Sağlık sistemi için AIR bildirimi önemlidir	1	2	3	4	5
7	FV alanında eğitim almış sağlık çalışanları AIR raporlamasında daha iyi bir rol oynar	1	2	3	4	5
8	Tüm sağlık çalışanlarının FV ve AIR raporlama sistemi hakkında eğitime ihtiyacı olduğuna inanıyormusunuz.	1	2	3	4	5
9	AIR'lerin ölümle sonuçlanabileceğini düşünüyor musunuz.	1	2	3	4	5

KKM: Kesinlikle katılmıyorum, KM:Katılmıyorum, B: Belirsiz, K: Katılıyorum, KK: Kesinlikle katılıyorum.

Bölüm 4. Lütfen aşağıdaki her bir soruya Evet (1) veya Hayır (2) olarak işaretleyiniz (Uygulama soruları).

No.	Sorular	Evet	Hayır
1	Karşılaştığınız AIR'yi hastanın klinik kaydına not ettiniz mi.	1	2
2	AIR kayıtlarını tutuyor musunuz.	1	2
3	FV irtibat noktasına veya ulusal farmakovijilans merkezine şüpheli bir AIR raporu gönderdiniz mi.	1	2
4	Hastanenizdeki sağlık çalışanları, AIR'yi nasıl bildireceklerikonusunda eğitildi mi.	1	2
5	Çalıştığınız kurumda AIR formlarına kolayca ulaşılabiliyor mu.	1	2
6	Hastalara ilaç vermeden önce her seferinde ilacın prospektüsünüokurum.	1	2
7	Hastalara her seferinde ilaç prospektüslerini okumalarını öneririm.	1	2
8	Hastalara her zaman ilaçların yan etkileri hakkında bilgi veririm.	1	2

Bölüm 5. Lütfen aşağıdaki her bir ifadeye ne ölçüde katıldığınızı veya katılmadığınızı belirti (Engeller soruları).

**No.**	**Sorular**	Evet	Hayır	Bilmiyorum
1	Çalıştığınız kurumda FV irtibat noktası bulunmaması mı?	1	2	3
2	FV merkezinden hastanelere eğitim desteği sağlanmıyor mu	1	2	3
3	Çalıştığınız kurumda AIR formları bulunmuyor mu.	1	2	3
4	AIR raporlamasında daha fazla zaman tüketeceğinizi mi düşünüyorsunuz.	1	2	3
5	Hastalarımın güvenini zedelemekten mi korkuyorsunuz.	1	2	3
6	AIR'leri tespit etmede bilgi yetersizliği mi sözkonusu.	1	2	3
7	AIR formları çok karmaşık mı.	1	2	3
8	Bildirilen AIR'nin yasal sorumluluğundan korkuyorum.	1	2	3
9	Rapor etmek için isteksizim.	1	2	3
10	Nasıl raporlama edeceğimi bilmiyorum.	1	2	3
11	AIR'yi saptamada farmakoterapi konusunda yetersiz bilgimin olması.	1	2	3
12	AIR'yi tartışmak için profesyonel ortamın olmaması.	1	2	3
13	Sağlık çalışanları üzerindeki yüksek iş yükü.	1	2	3
14	Sağlık yetkilileri tarafından yetersiz mali destek.	1	2	3
15	Unutkanlık bir engel mi.	1	2	3
16	Bir raporun bir fark yaratabileceğine inanıyormusunuz.	1	2	3
17	Diğer sağlık çalışanları AIR vakalarını bildirmiyor mu.	1	2	3
18	AIR raporlamasının bir göreviniz olmadığını mıdüşünyorsunuz	1	2	3

Zaman ayırdığınız ve değerlendirdiğiniz için teşekkürler.
