# Supplementary material for: Evaluation of health care professionals’ knowledge, attitudes, practices and barriers to pharmacovigilance and adverse drug reaction reporting: A cross-sectional multicentral study
Source: PLoS One. 2023 May 24;18(5):e0285811. doi: 10.1371/journal.pone.0285811 (PMC10208525; doi:10.1371/journal.pone.0285811)
Supplement: S1 Table — (DOCX) [file pone.0285811.s002.docx]

**S1 Table. Knowledge, attitude and practice score level among various profession of HCPs (Modified Bloom's cut-off point criteria for KAP score)**

| **KAP Level** | **Doctor (n=105)** | **Dentist**  **(n=38)** | **Pharmacist**  **(n=20)** | **Nurse**  **(n=150)** | **Midwife**  **(n=29)** | **Paramedic**  **(n=70)** | **Total**  **(n=412)** | ***P-value** |
| --- | --- | --- | --- | --- | --- | --- | --- | --- |
| **Knowledge Score** (Overall mean score=3.47 ±2.37, min-max = 0–8) | | | | | | | | **0.00** |
| 0 | 10 | 10 | 1 | 13 | 2 | 28 | 64 |  |
| 1 | 14 | 7 | 1 | 12 | 1 | 4 | 39 |  |
| 2 | 19 | 0 | 0 | 17 | 3 | 7 | 46 |  |
| 3 | 25 | 2 | 2 | 20 | 5 | 11 | 65 |  |
| **Poor** | **68 (64.7)** | **19 (50)** | **4 (20)** | **62 (41.3)** | **11 (37.9)** | **50 (71.4)** | **214 (51.9)** |  |
| 4 | 14 | 9 | 2 | 20 | 2 | 2 | 49 |  |
| 5 | 10 | 4 | 1 | 21 | 8 | 8 | 52 |  |
| **Moderate** | **24 (22.8)** | **13 (34.2)** | **3 (15)** | **41 (27.3)** | **10 (34.5)** | **10 (14.3)** | **101 (24.5)** |  |
| 6 | 5 | 5 | 3 | 27 | 5 | 4 | 49 |  |
| 7 | 5 | 0 | 4 | 12 | 3 | 5 | 29 |  |
| 8 | 3 | 1 | 6 | 8 | 0 | 1 | 19 |  |
| **Good** | **13 (12.4)** | **6 (15.8)** | **13 (65)** | **47 (31.3)** | **8 (27.6)** | **10 (14.3)** | **97 (23.5)** |  |
| **Attitude Score** (Overall mean score=38.37 ±7.305, min-max = 9-45). | | | | | | | | **0.00** |
| 9 | 0 | 0 | 0 | 3 | 1 | 1 | 5 |  |
| 11 | 1 | 0 | 0 | 2 | 0 | 0 | 3 |  |
| 12 | 0 | 0 | 0 | 0 | 0 | 1 | 1 |  |
| 21 | 2 | 0 | 0 | 0 | 0 | 0 | 2 |  |
| 22 | 0 | 2 | 0 | 1 | 0 | 0 | 3 |  |
| 25 | 0 | 0 | 0 | 1 | 0 | 0 | 1 |  |
| 26 | 0 | 0 | 0 | 0 | 0 | 1 | 1 |  |
| **Negative** | **3 (2.8)** | **2 (5.3)** | **0 (0)** | **7 (4.7)** | **1 (3.4)** | **3 (4.3)** | **16 (3.9)** |  |
| 27 | 2 | 4 | 0 | 6 | 2 | 9 | 23 |  |
| 28 | 1 | 5 | 0 | 0 | 0 | 0 | 6 |  |
| 29 | 1 | 0 | 0 | 6 | 0 | 1 | 8 |  |
| 30 | 0 | 0 | 0 | 0 | 0 | 1 | 1 |  |
| 31 | 2 | 0 | 0 | 1 | 1 | 1 | 5 |  |
| 32 | 1 | 0 | 0 | 6 | 0 | 2 | 9 |  |
| 33 | 4 | 0 | 0 | 5 | 0 | 3 | 12 |  |
| 34 | 3 | 1 | 0 | 4 | 0 | 2 | 10 |  |
| 35 | 3 | 3 | 1 | 17 | 2 | 3 | 29 |  |
| **Moderate** | **17 (16.2)** | **13 (34.2)** | **1 (5)** | **45 (30)** | **5 (17.2)** | **22 (31.4)** | **103 (25)** |  |
| 36 | 3 | 2 | 1 | 14 | 4 | 5 | 29 |  |
| 37 | 7 | 2 | 0 | 7 | 0 | 3 | 19 |  |
| 38 | 4 | 3 | 0 | 3 | 2 | 0 | 12 |  |
| 39 | 8 | 2 | 2 | 4 | 1 | 2 | 19 |  |
| 40 | 7 | 2 | 0 | 2 | 0 | 2 | 13 |  |
| 41 | 8 | 1 | 2 | 4 | 0 | 0 | 15 |  |
| 42 | 9 | 4 | 0 | 3 | 1 | 6 | 23 |  |
| 43 | 5 | 0 | 3 | 5 | 0 | 5 | 18 |  |
| 44 | 7 | 2 | 5 | 7 | 5 | 4 | 30 |  |
| 45 | 27 | 5 | 6 | 49 | 10 | 18 | 115 |  |
| **Positive** | **85 (81)** | **23 (60.5)** | **19 (95)** | **98 (65.3)** | **23 (79.3)** | **45 (64.3)** | **293 (71.1)** |  |
| **Practice Score** (Overall mean score=3.15±2.141, min-max = 9-45) | | | | | | | | **0.002** |
| 0 | 20 | 9 | 3 | 14 | 4 | 10 | 60 |  |
| 1 | 12 | 2 | 1 | 13 | 1 | 8 | 37 |  |
| 2 | 16 | 10 | 1 | 22 | 0 | 16 | 65 |  |
| 3 | 20 | 9 | 2 | 22 | 5 | 18 | 76 |  |
| 4 | 18 | 5 | 5 | 33 | 8 | 8 | 77 |  |
| 5 | 10 | 2 | 1 | 15 | 1 | 7 | 36 |  |
| 6 | 5 | 0 | 3 | 15 | 5 | 2 | 30 |  |
| **Poor** | **101 (96.2)** | **37 (97.4)** | **16 (80)** | **134 (89.3)** | **24 (82.8)** | **69 (98.6)** | **381 (92.5)** |  |
| 7 | 1 | 1 | 1 | 6 | 4 | 1 | 14 |  |
| 8 | 3 | 0 | 3 | 10 | 1 | 0 | 17 |  |
| **Good** | **4 (3.8)** | **1 (2.6)** | **4 (20)** | **16 (10.7)** | **5 (17.2)** | **1 (1.4)** | **31 (7.5)** |  |

± Standard deviation, Min=Minimum, Max=Maximum.
